# The prophase oocyte nucleus is a homeostatic G-actin buffer

**DOI:** 10.1242/jcs.259807

**Published:** 2022-03-23

**Authors:** Kathleen Scheffler, Federica Giannini, Tom Lemonnier, Binyam Mogessie

**Affiliations:** 1 School of Biochemistry, University of Bristol, Bristol BS8 1TD, UK; 2 Department of Molecular, Cellular and Developmental Biology, Yale University, New Haven, CT 06511, USA

**Keywords:** Oocyte, Nuclear F-actin, Meiosis, Aneuploidy, Chromosome segregation, Infertility

## Abstract

Formation of healthy mammalian eggs from oocytes requires specialised F-actin structures. F-actin disruption produces aneuploid eggs, which are a leading cause of human embryo deaths, genetic disorders and infertility. We found that oocytes contain prominent nuclear F-actin structures that are correlated with meiotic developmental capacity. We demonstrate that nuclear F-actin is a conserved feature of healthy mammalian oocytes and declines significantly with female reproductive ageing. Actin monomers used for nuclear F-actin assembly are sourced from an excess pool in the oocyte cytoplasm. Increasing monomeric G-actin transfer from the cytoplasm to the nucleus or directly enriching the nucleus with monomers led to assembly of stable nuclear F-actin bundles that significantly restrict chromatin mobility. By contrast, reducing G-actin monomer transfer by blocking nuclear import triggered assembly of a dense cytoplasmic F-actin network that is incompatible with healthy oocyte development. Overall, our data suggest that the large oocyte nucleus helps to maintain cytoplasmic F-actin organisation and that defects in this function are linked with reproductive age-related female infertility.

This article has an associated First Person interview with Federica Giannini, joint first author of the paper.

## Abstract (precis)

**Summary:** We demonstrate the existence of endogenous nuclear actin filaments in healthy mammalian oocytes, and that these F-actin structures deteriorate with advancing reproductive age.

## INTRODUCTION

Mammalian eggs are formed when oocyte chromosomes are segregated during meiosis (Mogessie et al., 2018), successful completion of which is a prerequisite for healthy embryogenesis and development. Meiotic chromosome segregation errors in oocytes are a leading cause of aneuploidies that underlie human infertility and genetic disorders, such as Down's syndrome (Herbert et al., 2015). The incidence of oocyte and egg aneuploidy increases almost exponentially with advancing female reproductive age (Hassold and Hunt, 2001; Nagaoka et al., 2012).

Distinct F-actin polymers assembled from soluble G-actin monomers ensure the production of healthy eggs from oocytes. These actin-rich drivers include a network of cytoplasmic actin filaments that oversee long-range vesicle transport (Schuh, 2011) and asymmetric division in mammalian oocytes (Holubcova et al., 2013; Schuh and Ellenberg, 2008; Azoury et al., 2008). In addition, oocyte meiotic spindles in several mammalian species contain actin filaments that aid microtubule fibres in chromosome separation (Mogessie and Schuh, 2017). After completion of meiosis, the actin cytoskeleton also plays a crucial role in pronuclear-stage zygotes, where it directly participates in the unification of parental genomes (Scheffler et al., 2021).

In this study, we show that actin filaments and bundles frequently occupy the nucleus in prophase-arrested mammalian oocytes. Furthermore, we demonstrate that, unlike in other experimental systems, endogenous nuclear actin filaments are assembled in healthy oocytes without any manipulation. By combining cytoskeletal disruption tools with nuclear import inhibition and high-resolution microscopy assays, we provided evidence for a homeostatic balance between cytoplasmic and nuclear F-actin structures in mammalian oocytes. Importantly, three-dimensional (3D) volumetric analyses of F-actin structures revealed that the amount and complexity of nuclear actin filaments declines significantly with increasing reproductive age in females.

## RESULTS

### Mammalian oocyte nuclei contain prominent F-actin structures

**Fig. 1.**
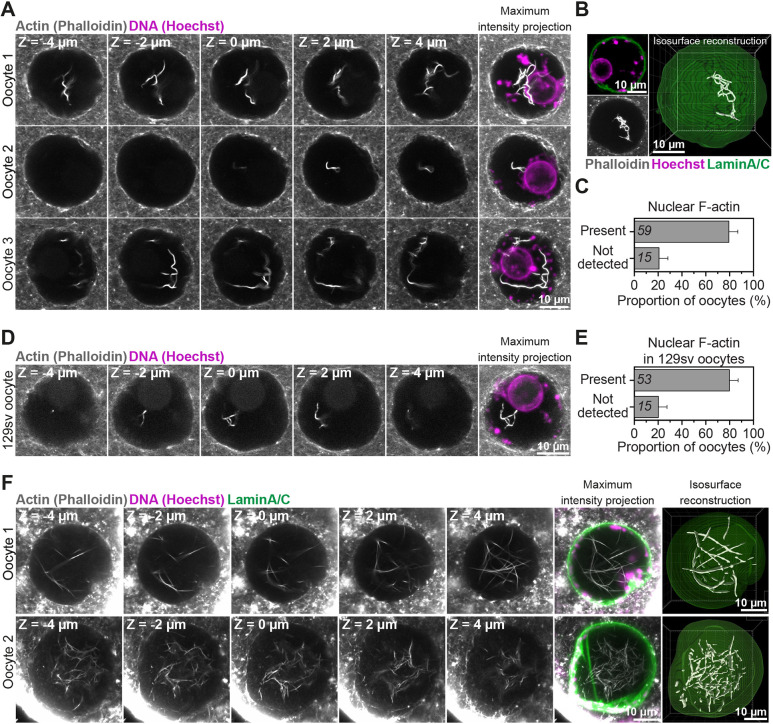
**Mammalian prophase oocyte nuclei contain prominent actin filaments.** (A) Phalloidin-labelled nuclear actin filaments (grey) and chromosomes (Hoechst, magenta) in three prophase-arrested mouse oocytes. Single confocal sections spaced 2 μm apart and corresponding maximum intensity projections are shown. (B) Pipeline for 3D isosurface reconstruction nuclear membrane (green) and nuclear F-actin (grey). DNA is shown in magenta. (C) Quantification of nuclear F-actin presence in prophase-arrested mouse oocytes. Data are from three independent experiments. (D) Phalloidin-labelled nuclear actin filaments (grey) and chromosomes (Hoechst, magenta) in prophase-arrested oocyte isolated from 129S6/SvEvTac mouse (inbred) strain. Single confocal sections spaced 2 μm apart and corresponding maximum intensity projections are shown. (E) Quantification of nuclear F-actin presence in prophase-arrested 129S6/SvEvTac strain mouse oocytes. Data are from three independent experiments. (F) Maximum intensity projection (nine confocal sections) images of phalloidin labelled nuclear F-actin (grey), DNA (magenta) and nuclear membrane (green) in two sheep oocytes. Single confocal sections spaced 2 μm apart are shown. Isosurface reconstruction of actin (white) demonstrates prominent nuclear actin filaments. Sheep oocytes from two independent experiments are shown.

**Fig. 2.**
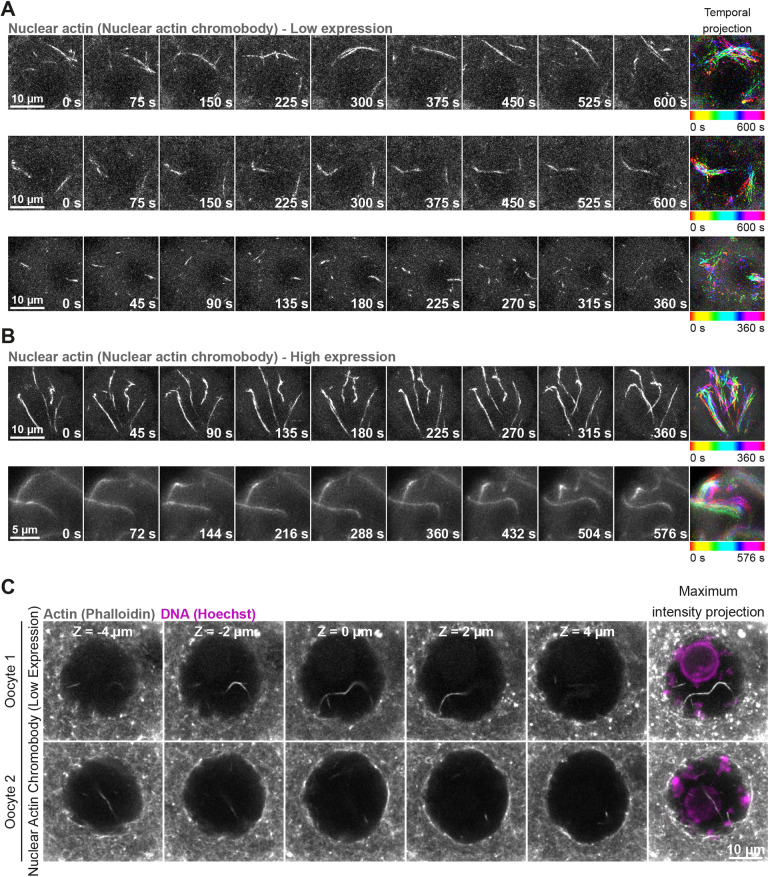
**Oocyte nuclear F-actin structures are dynamic.** (A) Super-resolution live imaging of nuclear actin filaments in three prophase-arrested mouse oocytes. Nuclear F-actin is labelled by low level expression of a fluorescent nanobody (nuclear actin chromobody). Colour-coded temporal projection images (built from 20 frames at 30 s intervals for top and middle panels, 25 frames at 15 s intervals for bottom panel) indicate mobility of filaments during the observation time. (B) Super-resolution live imaging of nuclear actin filaments in two prophase-arrested mouse oocytes. Nuclear F-actin is labelled by high level expression of a fluorescent nanobody (nuclear actin chromobody). Colour-coded temporal projection images (built from nine frames at 45 s intervals for top panel, 33 frames at 18 s intervals for bottom panel) indicate mobility of filaments during the observation time. (C) Phalloidin-labelled nuclear actin filaments (grey) and chromosomes (Hoechst, magenta) in two prophase-arrested mouse oocytes fixed after low-level expression and live imaging of nuclear actin chromobody. Single confocal sections spaced 2 μm apart and the corresponding maximum intensity projections are shown.

We have found that the nucleus of prophase-arrested, non-manipulated mouse oocytes contains prominent actin filaments (Fig. 1A). Using fluorescent phalloidin, we detected actin filaments and bundles in the nucleoplasm of 80% of fixed oocytes (Fig. 1B and C). Notably, this observation was strain- and species-independent, as we could detect nuclear F-actin structures in oocytes isolated from outbred and inbred mouse (Fig. 1D and E) and sheep ovaries (Fig. 1F). To visualise these structures in live cells, we microinjected prophase-arrested mouse oocytes with low to high concentrations of *in vitro* transcribed mRNAs encoding a fluorescence-labelled actin nanobody (nuclear actin chromobody) (Baarlink et al., 2017) and performed fast, super-resolution live imaging of nuclear F-actin. This approach revealed that these nuclear F-actin structures are highly mobile – filaments continuously move in non-directed fashion within the nucleoplasm (Fig. 2A and B; Movie 1 and Movie 2). To validate that low expression of the nuclear actin chromobody in these experiments did not grossly stabilise nuclear F-actin structures, we fixed microinjected oocytes after super-resolution live microscopy, labelled actin with fluorescent phalloidin and visualised the F-actin content of oocytes. This confirmed that nuclear F-actin structures in our live imaging experiments were not morphologically distinct from endogenous nuclear actin filaments (Fig. 1A and D, Fig. 2C). Importantly, the presence of nuclear F-actin was remarkably associated with distinct organisation of chromatin surrounding the nucleolus (Fig. S1A and B), a marker of high oocyte meiotic competence and developmental capacity (Zuccotti et al., 1998). These results collectively demonstrated that nuclear F-actin structures are a common feature of healthy, non-manipulated mammalian oocytes. Furthermore, we were able to detect nuclear F-actin structures by targeting F-tractin (Melak et al., 2017) and the calponin homology domain of utrophin (UtrCH) (Mogessie and Schuh, 2017) to the nucleus (Fig. S1C and D). These probes generally stabilised and reduced the mobility of nuclear actin filaments (Movies 3 and 4), indicating that they can be used to experimentally increase the F-actin content of mammalian oocyte nuclei.

### Cytoplasmic F-actin disruption induces excessive nuclear F-actin assembly

**Fig. 3.**
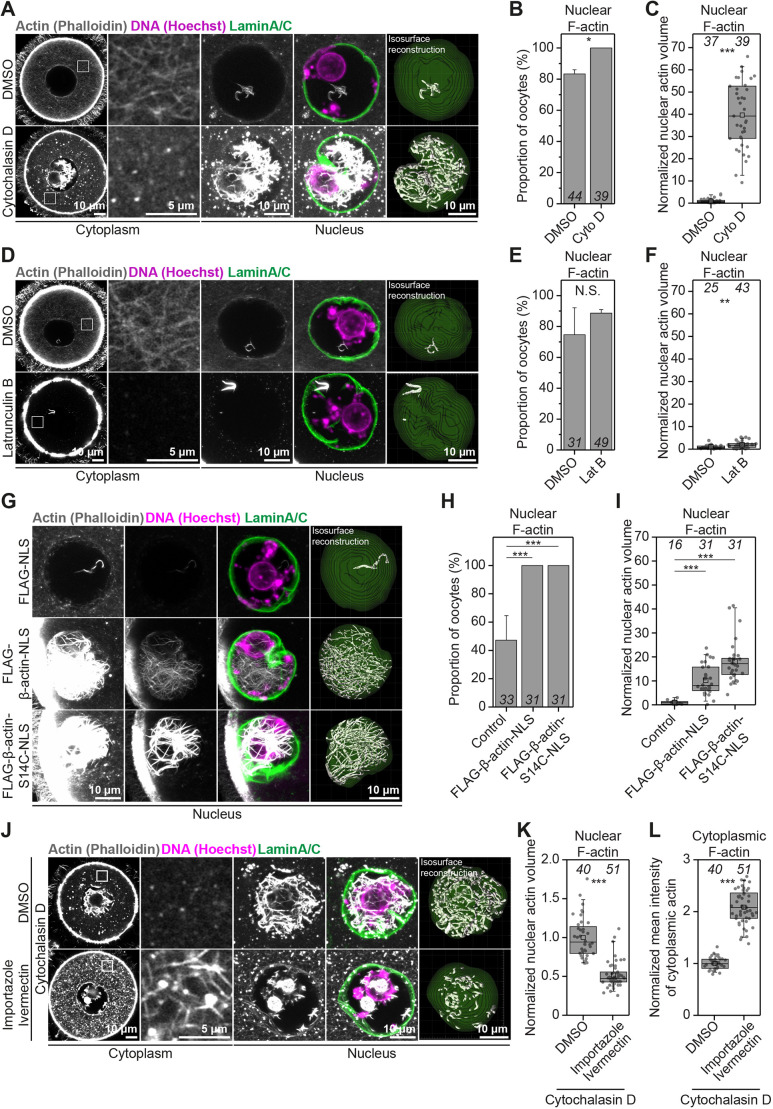
**Excess G-actin causes uncontrolled cytoplasmic and nuclear F-actin assembly.** (A) Single-section Airyscan images of phalloidin-labelled cytoplasmic F-actin and maximum intensity projections (nine confocal sections) of nuclear actin filaments (grey), DNA (magenta) and nuclear membrane (green) in DMSO- or Cytochalasin D-treated mouse oocytes. Boxed areas indicate regions within the oocyte cytoplasm that are shown magnified to the right of the respective Airyscan images. (B) Quantification of nuclear F-actin presence in DMSO- or Cytochalasin D-treated mouse oocytes. Data are from three independent experiments. (C) Quantification of nuclear F-actin volumes from isosurface reconstructions shown in A, in DMSO- or Cytochalasin D-treated mouse oocytes. Data are from three independent experiments. (D) Single-section Airyscan images of Phalloidin-labelled cytoplasmic F-actin and maximum intensity projections (nine confocal sections) of nuclear actin filaments (grey), DNA (magenta) and nuclear membrane (green) in DMSO- or Latrunculin B-treated mouse oocytes. Boxed areas indicate regions within the oocyte cytoplasm that are shown magnified to the right of the respective Airyscan images. (E) Quantification of nuclear F-actin presence in DMSO- or Latrunculin B-treated mouse oocytes. Data are from three independent experiments. (F) Quantification of nuclear F-actin volumes from isosurface reconstructions in D in DMSO- or Latrunculin B-treated mouse oocytes. Data are from three independent experiments. (G) Maximum intensity projection (nine confocal sections) images of phalloidin-labelled nuclear F-actin (grey), DNA (magenta) and nuclear membrane (green) in control and wild-type or S14C mutant actin expressing prophase-arrested oocytes. Excess nuclear F-actin in wild-type and S14C-overexpressing oocytes is demonstrated by presenting overexposed (when nuclear F-actin is highly visible in controls) or moderately overexposed (when nuclear F-actin is poorly visible in controls) images. (H) Quantification of nuclear F-actin presence in control and wild-type or S14C actin mutant-expressing mouse oocytes. Data are from three independent experiments. (I) Quantification of nuclear F-actin volumes from isosurface reconstructions in G in control and wild-type or S14C actin mutant-expressing mouse oocytes. Data are from three independent experiments. (J) Single-section Airyscan images of phalloidin-labelled cytoplasmic F-actin and maximum intensity projections (nine confocal sections) of nuclear actin filaments (grey), DNA (magenta) and nuclear membrane (green) in DMSO- or Importazole/Ivermectin-treated mouse oocytes that were then treated with Cytochalasin D. Boxed areas indicate regions within the oocyte cytoplasm that are shown magnified to the right of the respective Airyscan images. (K) Quantification of nuclear F-actin volumes from isosurface reconstructions in J of DMSO- or Importazole/Ivermectin-treated mouse oocytes that were then treated with Cytochalasin D. Data are from three independent experiments. (L) Quantification of cytoplasmic F-actin network intensity in DMSO- or Importazole/Ivermectin-treated mouse oocytes that were then treated with Cytochalasin D. Data are from three independent experiments. Statistical significance was tested using Fisher's exact test (B, E and H) and two-tailed Student's *t*-test (C, F, I, K and L).

We next sought to disrupt nuclear F-actin structures by using cytoskeletal drugs. Unexpectedly, we observed that disruption of the oocyte cytoplasmic actin network by Cytochalasin D (Schuh, 2011) treatment, instead, triggered excessive nuclear F-actin assembly (Fig. 3A). Nuclei were also more likely to contain F-actin structures when oocytes were treated with Cytochalasin D (Fig. 3B). To confirm this, we imaged fluorescent phalloidin-labelled nuclear actin filaments by high-resolution microscopy, selectively reconstructed them in 3D and quantified their volume inside nuclei – marked with antibodies against nuclear membrane proteins – of oocytes treated with DMSO (Control) or Cytochalasin D (Fig. 3A). This showed a near fortyfold increase in nuclear F-actin volume after disruption of the cytoplasmic actin network (Fig. 3C). Actin polymerises into filaments when monomer concentration exceeds critical concentration (Doolittle et al., 2013). We therefore reasoned that bulk transfer of Cytochalasin D-generated G-actin monomers from a large oocyte cytoplasm to a smaller nuclear volume could drive excess F-actin polymerisation. Such filaments are likely to be drug resistant because Cytochalasin D is generally less effective at high actin monomer concentration (Carlier et al., 1986). We tested this possibility by using Latrunculin, which disrupts the cytoplasmic actin network by sequestering monomers and preventing their addition to actin filaments, to complement our Cytochalasin D studies in a mechanistically distinct manner (Fig. 3D) (Spector et al., 1983; Coue et al., 1987; Morton et al., 2000). Under this circumstance, more cytosolic monomers would still be transferred to the nucleus but would be unable to participate in actin polymerisation. In stark contrast to treatment with Cytochalasin D, nuclei in Latrunculin B-treated oocytes were not more likely to contain actin filaments (Fig. 3E) and only showed a twofold increase in the volume of F-actin (Fig. 3F; Fig. S2A). Thus, the concentration of polymerisation-ready monomers imported from the cytoplasm is likely to determine the degree of F-actin assembly in the oocyte nucleus. Consistent with these perturbations, treatment of oocytes with the Arp2/3 inhibitor compound CK-666 also significantly increased polymerisation of nuclear F-actin (Fig. S2B and C).

We next took an approach to directly observe the transfer of excess actin monomers from the cytoplasm to the nucleus. For this, we first overexpressed SNAP-tagged beta-actin (SNAP-beta-actin) in prophase-arrested mouse oocytes. We then performed high-resolution time-lapse microscopy to assess the fluorescence of cytoplasmic and nuclear beta-actin immediately after addition of Cytochalasin D to the oocyte culture medium. This live imaging experiment revealed that cytoplasmic fluorescence of beta-actin rapidly decreased after Cytochalasin D addition, while the nuclear fluorescence signal increased notably before the nuclear assembly of F-actin bundles (Fig. S2D, Movie 5). Excess amount of cytoplasmic actin monomers can therefore be transferred to the oocyte nucleus, where they participate in nuclear F-actin polymerisation. Consistent with our data, increasing cellular G-actin by using cytoskeletal drugs in frog egg extracts (Parisis et al., 2017) or in cultured mammalian cells (Pendleton et al., 2003) can cause nuclear F-actin polymerisation. Excess transfer of cytosolic actin to the nucleus might thus be an actin homeostasis feature that is conserved in other cell types.

We next directly tested whether high monomeric G-actin concentration is sufficient to induce F-actin polymerisation in oocytes. We exceeded the nuclear monomeric G-actin concentration in mouse oocytes by targeting FLAG-tagged beta-actin (FLAG-beta-actin) to the nucleus by using a nuclear localisation signal (NLS). This led to a tenfold increase in the volume of nuclear F-actin in FLAG-beta-actin-NLS expressing oocytes, which were also more likely to contain F-actin than FLAG-NLS-expressing Control oocytes (Fig. 3G–I). Therefore, the concentration of monomeric G-actin in the nucleus indeed dictates the extent of nuclear F-actin polymerisation.

### Maintenance of oocyte cytoplasmic F-actin network organisation requires nuclear import activity

To further examine the transfer of G-actin monomers to the nucleus, we blocked nuclear import by using a combination of Importazole and Ivermectin (Soderholm et al., 2011; Wagstaff et al., 2012) (Fig. S3A), before disrupting the cytoplasmic actin network. Treatment of oocytes with Cytochalasin D did not cause excessive nuclear F-actin assembly when nuclear import was blocked (Fig. 3C,J,K; Fig. S3B). In addition, nuclear import blockage caused distinct accumulation of actin on the surface of oocyte nuclei (Fig. S3D). Surprisingly, Cytochalasin D treatment of oocytes defective in nuclear import resulted in a significantly denser cytoplasmic actin network, and one that was composed of drug-resistant filaments (Fig. 3J,L; Fig. S3C). This is consistent with a significant rise in cytosolic monomeric G-actin concentration caused by blocked nuclear import, which reduces Cytochalasin D activity (Carlier et al., 1986) in the cytoplasm and causes assembly of drug-resistant filaments. Prompt transfer of excess G-actin monomers to the nucleus could be necessary to maintain the organisation of cytoplasmic F-actin network in oocytes. This is supported by the observation that blocking nuclear import in oocytes not previously treated with cytoskeletal drugs significantly increases cytoplasmic actin network density (Fig. S3D and E). Indeed, monomeric actin is known to continuously shuttle between the cytoplasm and the nucleus in other experimental systems (Dopie et al., 2012; Stuven et al., 2003). Thus, notwithstanding the role of other nuclear import cargoes in these observations, our data support the notion that excess actin monomer import helps to maintain cytoplasmic F-actin network organisation in mouse oocytes. We propose that shuttling of cytosolic monomers into a large (∼30 µm diameter) nucleus is a physiological G-actin-buffering process that oocytes use to continuously modulate the cytoplasmic actin network.

### Stable nuclear actin filaments restrict chromatin mobility in oocytes

**Fig. 4.**
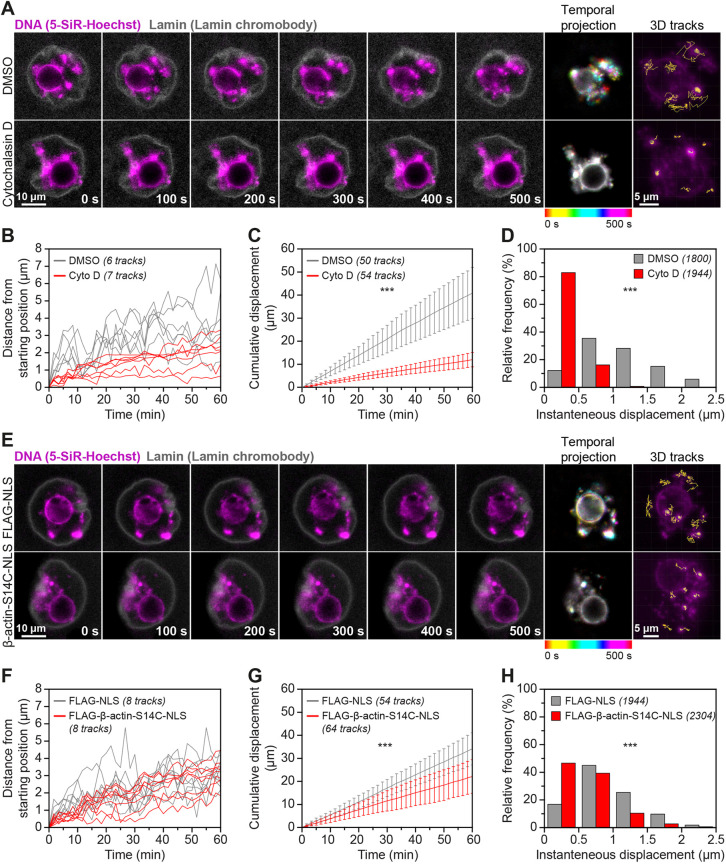
**Excess nuclear actin filaments severely restrict oocyte chromatin mobility.** (A) Still images from representative time-lapse movies of chromatin movement in DMSO- or Cytochalasin D-treated mouse oocytes. Chromatin (SiR-5-Hoechst) is shown in magenta and nuclear membrane (lamin chromobody) is shown in grey. Colour-coded temporal projection images indicate the degree of chromatin mobility during the 500 s observation time. 3D tracks represent the spatial coverage of prominent chromatin spots over a 60-min observation period. (B) Distance from starting position of prominent chromatin spots in 3D over a 60-min observation period in DMSO- or Cytochalasin D-treated mouse oocytes. Data are from three independent experiments. (C) Cumulative instantaneous displacement of prominent chromatin spots in 3D over a 60-min observation period in DMSO- or Cytochalasin D-treated mouse oocytes. Data are from three independent experiments. Two-way analysis of variance was used to test for significance. (D) Relative frequencies of chromatin spot instantaneous displacement in DMSO- or Cytochalasin D-treated mouse oocytes. Data are from three independent experiments. Two-tailed Student's *t*-test was used to evaluate statistical significance. (E) Still images from representative time-lapse movies of chromatin movement in control or S14C actin mutant expressing mouse oocytes. Chromatin (SiR-5-Hoechst) is shown in magenta and nuclear membrane (Lamin chromobody) is shown in grey. Colour-coded temporal projection images indicate the degree of chromatin mobility in the 500 s observation time. 3D tracks represent the spatial coverage of prominent chromatin spots over a 60-min observation period. (F) Distance from starting position of prominent chromatin spots in 3D over a 60-min observation period in control or S14C actin mutant expressing mouse oocytes. Data are from three independent experiments. (G) Cumulative instantaneous displacement of prominent chromatin spots in 3D over a 60-min observation period in control or S14C actin mutant expressing mouse oocytes. Data are from three independent experiments. Two-way analysis of variance was used to evaluate statistical significance. (H) Relative frequencies of chromatin spot instantaneous displacement in control or S14C actin mutant expressing mouse oocytes. Data are from three independent experiments. Two-tailed Student's *t*-test was used to evaluate statistical significance.

To investigate the consequences of stable assembly of nuclear actin filaments, we induced excess cytosolic monomers by treating oocytes with Cytochalasin D (Fig. 3A) or directly enriched oocyte nuclei with monomers by expressing a nuclear actin mutant (FLAG-NLS-beta-actin-S14C) that is more able to polymerise (Posern et al., 2004; Wei et al., 2020) (Fig. 3G–I). We then visualised chromatin (by staining with 5-SiR-Hoechst) and the nuclear envelope (by using fluorescent nuclear membrane nanobodies) at high-temporal resolution. Initial analysis of these data indicated that excess nuclear actin filaments led to notably reduced chromatin mobility (Fig. 4A and E; Movies 6–9). We investigated this further by automated 3D tracking of prominent chromatin spots throughout the nucleoplasm (Fig. 4A and E). Indeed, nuclei containing excess levels of F-actin showed significantly less movement of chromatin spots over time (Fig. 4B–D,F–H). These data are consistent with recent observations that Cytochalasin D treatment dampens chromatin mobility in mouse oocytes (Almonacid et al., 2019).

**Fig. 5.**
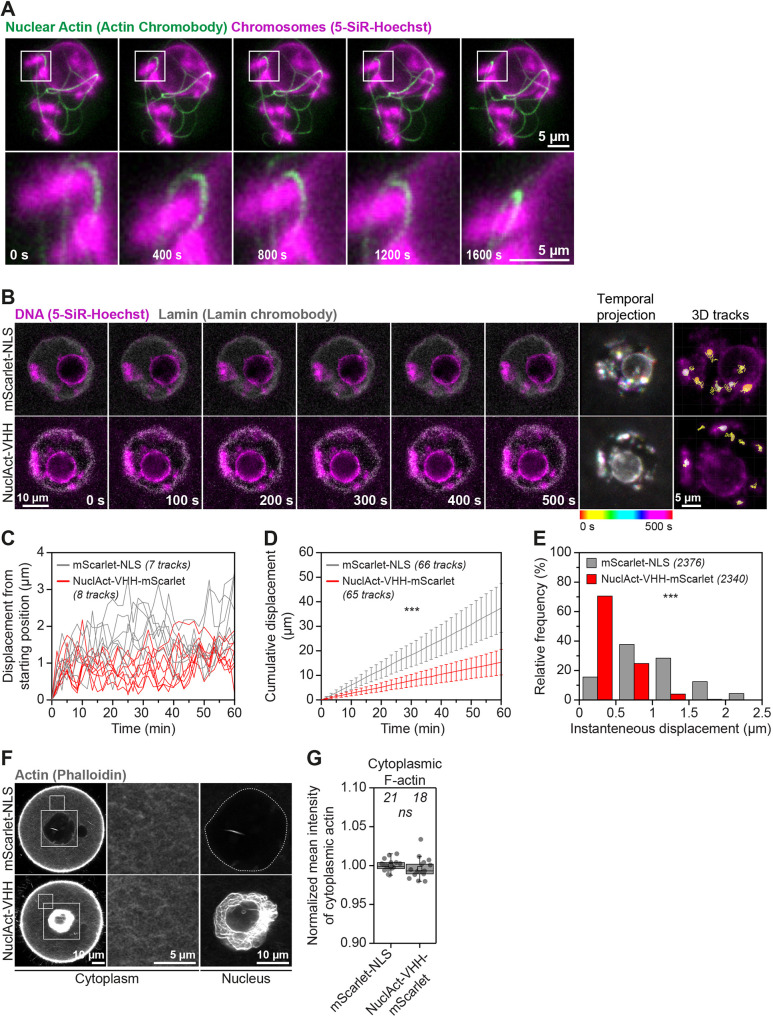
**Targeted nuclear F-actin stabilisation diminishes chromatin mobility.** (A) Still images from a time-lapse movie of chromatin (5-SiR-Hoechst, magenta) and stabilised nuclear F-actin (overexpressed nuclear actin chromobody, green). Boxed areas are shown magnified in images below. (B) Still images from representative time-lapse movies of chromatin movement in control (mScarlet-NLS) or nuclear actin chromobody overexpressing (NuclAct-VHH) mouse oocytes. Chromatin (SiR-5-Hoechst) is shown in magenta and nuclear membrane (Lamin chromobody) is shown in grey. Colour-coded temporal projection images indicate the degree of chromatin mobility in the 500 s observation time. 3D tracks represent the spatial coverage of prominent chromatin spots over a 60-min observation period. (C) Distance from starting position of prominent chromatin spots in 3D over a 60-min observation period in control (mScarlet-NLS) or nuclear actin nanobody- (NuclAct-VHH-mScarlet) overexpressing mouse oocytes. Data are from three independent experiments. (D) Cumulative instantaneous displacement of prominent chromatin spots in 3D over a 60-min observation period in control (mScarlet-NLS) or nuclear actin nanobody (NuclAct-VHH-mScarlet) overexpressing mouse oocytes. Data are from three independent experiments. Two-way analysis of variance was used to evaluate statistical significance. (E) Relative frequencies of chromatin spot instantaneous displacement in control (mScarlet-NLS) or nuclear actin nanobody- (NuclAct-VHH-mScarlet) overexpressing mouse oocytes. Data are from three independent experiments. Two-tailed Student's *t*-test was used to evaluate statistical significance. (F) Single-section Airyscan images of phalloidin-labelled cytoplasmic F-actin and maximum intensity projections (nine confocal sections) of nuclear actin filaments (grey) in Control (mScarlet-NLS) or nuclear actin nanobody- (NuclAct-VHH-mScarlet) overexpressing mouse oocytes. Boxes in the oocyte cytoplasm and surrounding the nucleus mark regions that are magnified in insets. (G) Quantification of cytoplasmic F-actin network intensity in Control (mScarlet-NLS) or nuclear actin nanobody- (NuclAct-VHH-mScarlet) overexpressing mouse oocytes. Data are from three independent experiments. Statistical significance was evaluated using two-tailed Student's *t*-test.

Our results strongly indicated that reduced chromatin movement in cytoskeletal perturbation assays are more attributed to increased nuclear F-actin levels than with disruption of the cytoplasmic actin networks. This prediction was supported by live imaging experiments, revealing that stable nuclear actin filaments (induced by high expression of nuclear actin chromobody) can physically entrap chromatin (marked with histone H2B) (Fig. 5A; Movie 10). We therefore used this nuclear actin chromobody to stabilise F-actin inside the oocyte nucleus and track the mobility of chromatin in 3D. Consistent with our Cytochalasin D addition and nuclear-specific actin monomer enrichment experiments, stabilisation of nuclear F-actin by using this approach diminished chromatin movement (Fig. 5B–E; Movies 11 and 12). Importantly, this manipulation did not alter cytoplasmic F-actin networks (Fig. 5F and G). These results demonstrate that nuclear F-actin stabilisation is sufficient to restrict chromatin movement (Fig. 3I–K; Movies 11 and 12) and provide new explanation for why drugs that disrupt cytoplasmic F-actin also impact chromatin mobility. Dynamic movement of chromatin within the nucleus has recently been linked to basal level of transcription in mouse oocytes (Almonacid et al., 2019). We thus expect stable nuclear F-actin bundles that arise from excess monomer import and drastically reduce chromatin mobility to ultimately impact transcriptional dynamics in oocytes. Induction of stable nuclear actin filaments in cultured cells that do not normally contain nuclear F-actin has also been shown to inhibit transcription by RNA polymerase II (Serebryannyy et al., 2016a). However, the underlying mechanisms in this model are thought to involve sequestration of nuclear actin monomers that are actively imported and support transcription through interactions between actin and RNA polymerase II (Dopie et al., 2012; Serebryannyy et al., 2016a).

### Actin mutants severely impact oocyte chromosomal alignment and segregation

**Fig. 6.**
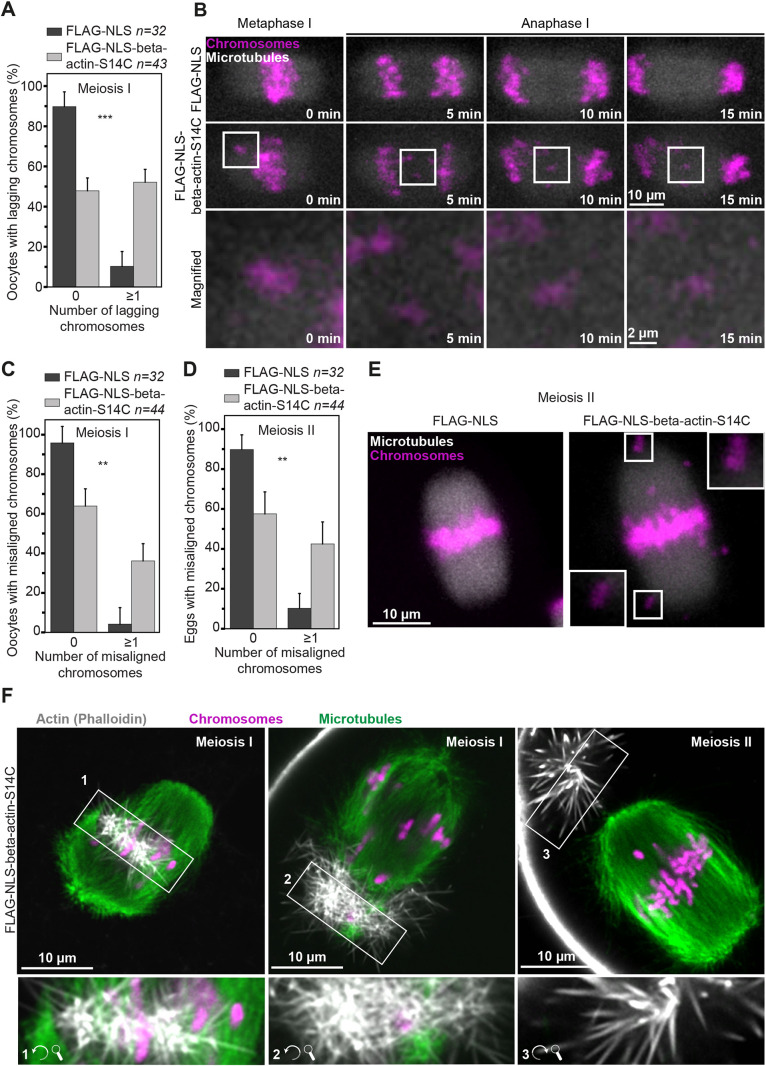
**Actin mutants that disrupt nuclear dynamics compromise oocyte meiosis.** (A) Frequency of lagging chromosomes in control- (optimal nuclear F-actin in prophase) and S14C actin mutant-expressing (excess nuclear F-actin in prophase) mouse oocytes. Data are from four independent experiments. (B) Still images from representative time-lapse movies of chromosome segregation during meiosis I in control or S14C actin mutant-expressing oocytes. Microtubules (EGFP-MAP4-MTBD) are shown in grey and chromosomes (H2B-mRFP) are shown in magenta. Boxed areas are shown magnified in bottom panels. (C) Frequency of misaligned chromosomes during meiosis I in control (optimal nuclear F-actin in prophase) and S14C actin mutant-expressing (excess nuclear F-actin in prophase) mouse oocytes. Data are from four independent experiments. (D) Frequency of misaligned chromosomes during meiosis II in control (optimal nuclear F-actin in prophase) and S14C actin mutant-expressing (excess nuclear F-actin in prophase) mouse eggs. Data are from four independent experiments. (E) Representative images of fully aligned chromosomes in control (FLAG-NLS) and severely misaligned chromosomes (white arrows) in S14C actin mutant-expressing mouse eggs. Microtubules (EGFP-MAP4-MTBD) are shown in grey, chromosomes (H2B-mRFP) are shown in magenta. Boxed areas are shown in insets magnified (5×). (F) Single-section Airyscan images of actin (grey), microtubules (green) and chromosomes (magenta) in S14C actin mutant-expressing (excess nuclear F-actin in prophase) oocytes during meiosis I and in one egg during meiosis II. Numbered and boxed areas indicate stable actin filament bundles that are shown rotated and magnified (2×) in the insets below. Statistical significance was evaluated in A, C and D by using Fisher's exact test.

Importantly, some disease-causing actin mutants also lead to assembly of persistent nuclear actin filaments in interphase nuclei that typically do not contain F-actin (Serebryannyy et al., 2016b). In this context, we probed the lasting effect of nuclear beta-actin-S14C mutants on oocyte meiosis by live imaging of metaphase chromosome dynamics in oocytes that contained excess nuclear F-actin bundles during meiotic prophase arrest. These experiments revealed striking defects in chromosome alignment and segregation in oocytes expressing mutant beta-actin-S14C. Compared to ∼90% of control oocytes that progressed normally through meiosis I, ∼50% of oocytes expressing actin mutants showed at least one lagging chromosome during anaphase I (Fig. 6A and B; Movies 13 and 14). In addition, expression of the 14C actin mutant caused significant metaphase chromosome misalignment in oocytes and metaphase II-arrested eggs (Fig. 6C–E; Movies 13 and 14). Super-resolution immunofluorescence microscopy of F-actin, microtubules and chromosomes in oocytes expressing actin mutants further suggested that, after nuclear envelope disassembly, stable F-actin structures become embedded in meiotic spindles where they are likely to obstruct chromosomal organisation (Fig. 6F).

### Targeted actin mutant degradation restores meiotic fidelity in oocytes

**Fig. 7.**
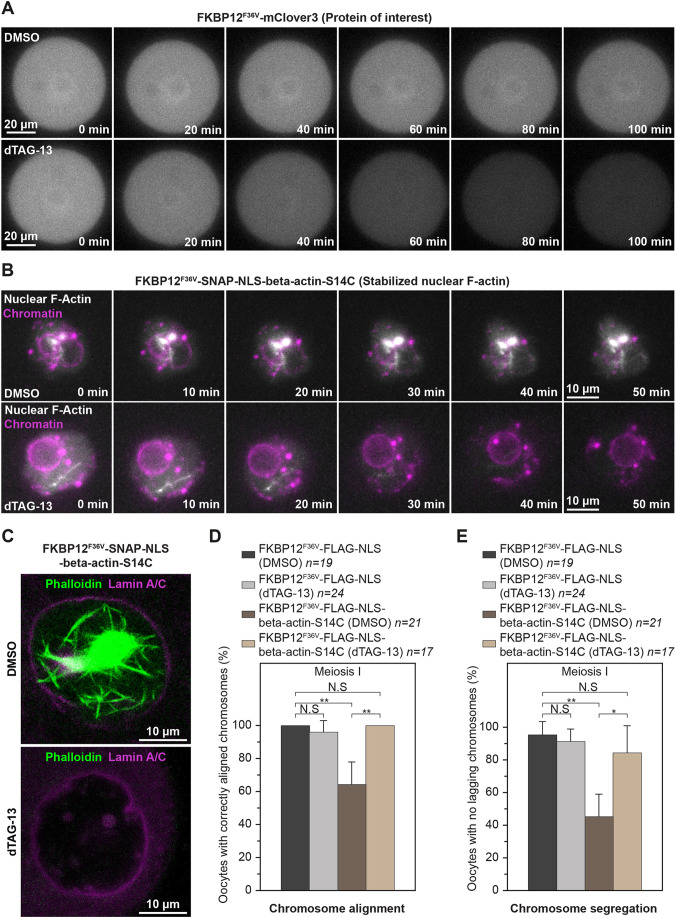
**Targeted degradation of stable nuclear actin filaments restores chromosome segregation fidelity in oocytes.** (A) Panels from time-lapse movies of Control (DMSO) and dTAG-13-treated prophase-arrested mouse oocytes expressing FKBP12^F36V^-mClover3. (B) Rapid degradation of oocyte nuclear F-actin bundles using the dTAG system. Panels from time-lapse movies of Control (DMSO) and dTAG-13-treated prophase-arrested mouse oocytes expressing FKBP12^F36V^-SNAP-NLS-beta-actin-S14C are shown. (C) Phalloidin labelling of nuclear F-actin in FKBP12^F36V^-SNAP-NLS-beta-actin-S14C expressing prophase-arrested mouse oocytes fixed after treatment with DMSO (Control) (DMSO) or dTAG-13. (D) Quantification of metaphase I oocytes expressing Control (FKBP12^F36V^-FLAG-NLS) or FKBP12^F36V^-FLAG-NLS-beta-actin-S14C with correctly aligned chromosomes after maturation in DMSO or dTAG-13 (to degrade actin mutants). Data are from three independent experiments. (E) Quantification of metaphase I oocytes expressing Control (FKBP12^F36V^-FLAG-NLS) or FKBP12^F36V^-FLAG-NLS-beta-actin-S14C with correctly segregated chromosomes after maturation in DMSO or dTAG-13 (to degrade actin mutants). Data are from three independent experiments. Statistical significance was assessed using Fisher's exact test in D and E.

To directly confirm whether chromosome alignment and separation defects arise from actin mutants that are retained in the cytoplasm after nuclear envelope disassembly, we sought to rapidly degrade these mutants after releasing oocytes from prophase arrest. We thus adapted the degradation tag (dTAG) system, a recently innovated method for targeted and rapid degradation of FKBP12^F36V^-tagged proteins (Nabet et al., 2018, 2020) (Fig. S4A), for use in mouse oocytes. Our proof-of-concept experiments using the fluorescent protein mClover3 showed that the small molecule dTAG-13 (see Materials and Methods) can degrade overexpressed FKBP12^F36V^-mClover3 within 100 min of addition (Fig. 7A; Movies 15 and 16). Live imaging experiments further demonstrated that dTAG-13 is highly efficient in rapidly degrading SNAP-NLS-FKBP12^F36V^-beta-actin-S14C mutants, thereby effectively eliminating stable nuclear F-actin cables as early as 40 min after the addition of small molecules (Fig. 7B; Movies 17 and 18). Phalloidin staining of DMSO- and dTAG-13-treated oocytes confirmed the complete degradation of stable nuclear F-actin bundles when this approach had been used (Fig. 7C). We therefore generated and expressed FKBP12^F36V^-FLAG-NLS (Control) and FKBP12^F36V^-FLAG-NLS-beta-actin-S14C mRNA in prophase-arrested oocytes. Consistent with our previous experiments, this led to chromosome alignment and segregation errors in oocytes that contained S14C mutants (Fig. 7D and E; Movies 19 and 20). dTAG-mediated degradation of actin mutants before meiotic spindle assembly successfully rescued defects of chromosome dynamics in oocytes that expressed FKBP12^F36V^-FLAG-NLS-beta-actin-S14C (Fig. 7D and E; Movies 21 and 22). This confirmed that actin mutants that disrupt nuclear dynamics can also compromise later stages of mammalian oocyte meiosis. Future studies that explore physiologically relevant actin mutants in the context of oocyte meiosis could therefore advance the current understanding of causes of female infertility.

## DISCUSSION

**Fig. 8.**
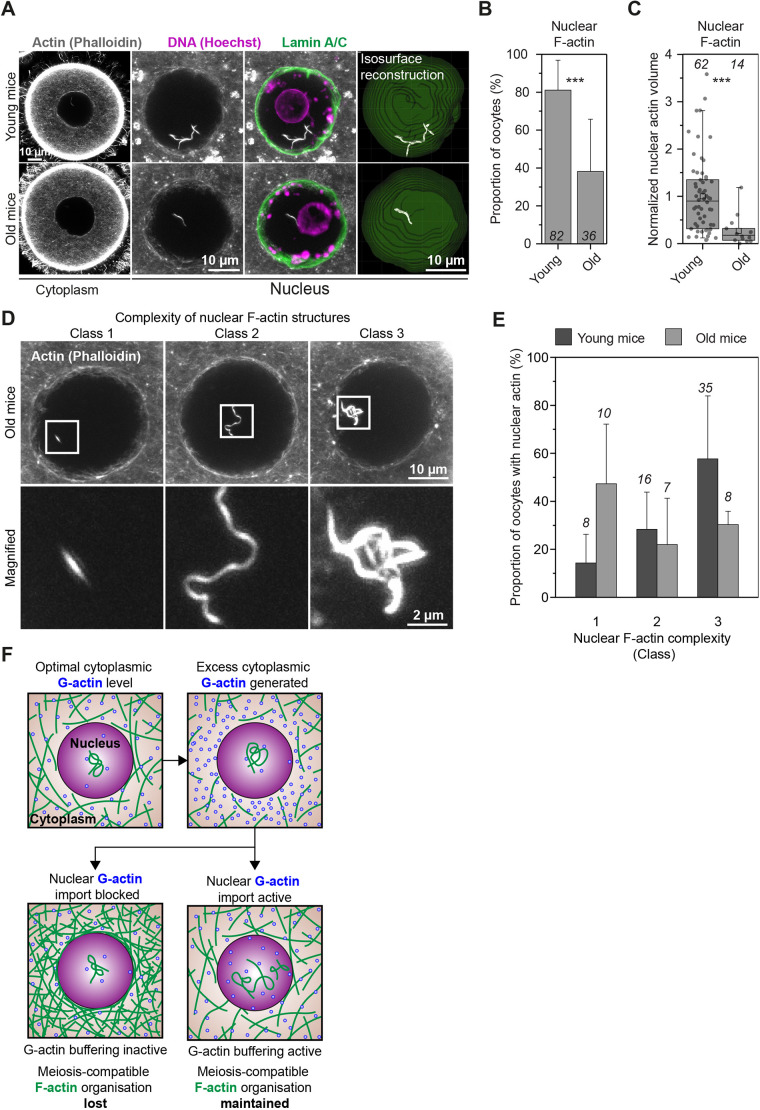
**Oocyte nuclear F-actin abundance and complexity declines with female reproductive age.** (A) Single-section Airyscan images of phalloidin-labelled cytoplasmic F-actin and maximum intensity projections (nine confocal sections) of nuclear actin filaments (grey), DNA (magenta) and nuclear membrane (green) in oocytes isolated from young and old mice. (B) Quantification of nuclear F-actin presence in oocytes isolated from young and old mice. Data are from three independent experiments. (C) Quantification of nuclear F-actin volumes from isosurface reconstructions in E in oocytes isolated from young and old mice. Data are from three independent experiments. (D) Three representative classes of nuclear F-actin (grey) complexity in phalloidin-labelled mouse oocytes. Boxes mark regions magnified in bottom panels. (E) Quantification of the different classes of nuclear F-actin complexity (shown in D) in oocytes isolated from young and old mice. Data are from three independent experiments. (F) Model for the regulation of cytoplasmic F-actin organisation when using a homeostatic G-actin buffer. When G-actin buffering is active, excess cytosolic G-actin monomers are promptly transferred into the large oocyte nucleus. When G-actin buffering is inactive, cytosolic G-actin concentration increases. This leads to assembly of a dense cytoplasmic actin network that interferes with the formation of healthy eggs. Statistical significance was evaluated using Fisher's exact test (B) and two-tailed Student's *t*-test (C).

Transferring excess monomeric G-actin to the nucleus could be physiologically important when prophase-arrested oocytes resume meiosis, en route to becoming eggs. This process is accompanied by a natural reduction in cytoplasmic F-actin network density that is thought to support asymmetric cell division (Holubcova et al., 2013). It is conceivable that such F-actin reduction, similarly to drug-mediated F-actin disruption, also releases actin monomers into the cytoplasm. Prompt monomer transfer to the large nucleus might therefore enable oocytes to maintain a cytoplasmic F-actin organisation that is compatible with healthy meiosis. Importantly, we found that the amount and complexity of nuclear F-actin structures in non-manipulated mouse oocytes significantly declines with increasing reproductive age in females – with oocytes from 12-month-old mothers having only 27% of the nuclear F-actin levels observed in younger (8–12 weeks old) mothers (Fig. 8A–E). This raises the intriguing possibility that loss of nuclear actin polymerisation capacity in oocytes could be linked with reproductive age-related female infertility.

Homeostatic G-actin buffering (Fig. 8F) could be a widely conserved feature of large mammalian oocyte nuclei. For instance, prophase nuclei in non-manipulated sheep oocytes can also contain prominent nuclear actin filaments (Fig. 1F). In frog oocytes, where the nucleus measures over 400 μm in diameter, F-actin maintains organisation of the nucleus by stabilising its contents against gravitational forces (Feric and Brangwynne, 2013). Our data therefore extend to mammalian experimental models the observation that F-actin is linked to the dynamics of large nuclei. Interestingly, nuclear F-actin is also known to assemble in a variety of cellular contexts in non-gamete cells and embryos (Baarlink et al., 2017, 2013; Caridi et al., 2019; Plessner et al., 2015; Wang et al., 2019; Kelpsch and Tootle, 2018; Wesolowska and Lenart, 2015; Okuno et al., 2020) with postulated functions ranging from DNA repair to chromatin organisation. It will be important to explore whether the oocyte G-actin buffering process we described here is a universal feature of mammalian nuclei and non-mammalian models in which F-actin structures are intimately associated with the nucleus (Mori et al., 2014; Wesolowska et al., 2020). In addition, mechanotransduction of actin-based forces to the nucleus is known to modulate nuclear mechanics and function in health and disease (Isermann and Lammerding, 2013; Martino et al., 2018; Alisafaei et al., 2019). However, a direct role of the nucleus itself in this process by regulating cytosolic G-actin concentration, and thus F-actin assembly and force generation, should now be considered. Finally, our data indicate that commonly used actin drugs unintendedly stabilise nuclear F-actin and significantly affect nuclear mechanics. This will have important implications for past and future studies of sub-cellular actin-based structures in single- and multi-nucleated cells.

## MATERIALS AND METHODS

### Preparation and microinjection of mammalian oocytes

All mice were maintained in a specific pathogen-free environment according to UK Home Office regulations and the guidelines of the University of Bristol Animal Services Unit. Oocytes were isolated from ovaries of 129S6/SvEvTac mice aged between 8 and 12 weeks (young CD-1 mice), 10 and 12 months (old CD-1 mice) or 8 and 12 weeks, cultured and microinjected with 6–8 pl of *in vitro* transcribed mRNA, as described in detail recently (Mogessie, 2020).

Sheep ovaries were obtained from a University of Bristol Veterinary School slaughterhouse and transported to the laboratory, while stored at 37°C in M2 medium (Mogessie, 2020). Oocytes covered with several layers of cumulus cells were collected from ovaries by aspiration with an 18-gauge needle and cultured in M2 medium supplemented with 750 μM N6,2′-O-dibutyryladenosine 3′,5′-cyclic monophosphate sodium salt (dbcAMP) before fixation and processing.

### Generation of expression constructs and mRNA synthesis

To mark microtubules, an EGFP variant of the microtubule binding domain of mouse MAP4 (MAP4-MTBD, 659–1125 aa) (Mogessie et al., 2015) was generated as described by Mogessie and Schuh (2017). To label chromosomes, the coding sequence of histone H2B was obtained from mouse cDNA and transferred by using Gibson assembly into the HindIII site of pmRFP-N3 using primers 5′-GGACTCAGATCTCGAGCTCAATGCCTGAGCCTGCGAAG-3′ and 5′-CCGTCGACTGCAGAATTCGACTTGGAGCTGGTGTACTTGG-3′. The fragment corresponding to H2B-mRFP was then transferred into the XhoI-NotI site of pGEMHE (Schuh and Ellenberg, 2007) to generate the final construct pGEM-H2B-mRFP. To label nuclear actin, fluorescent nuclear actin nanobody (nuclear actin chromobody) plasmid was purchased from ChromoTek and transferred into the HindIII-EcoRI site of pGEMHE. To label the nuclear envelope, fluorescent lamin nanobody (lamin chromobody) plasmid was purchased from ChromoTek and transferred into the NcoI-XbaI site of pGEMHE. Wild-type and S14C actin mutant expression constructs were generated using a synthetic construct encoding the SV40 nuclear localisation signal (NLS) (5′-CCGCCTAAGAAAAAGCGGAAGGTG-3′) fused to mouse beta-actin (NM_007393.5). pGEM-FLAG-NLS beta-actin was generated by PCR linearisation of pGEMHE with primers 5′-AATTCTGCAGTCGACGGC-3′ and 5′-CGAAGCTTGAGCTCGAGATC-3′ and joined by using Gibson assembly with NLS-beta-actin that was flanked by primers 5′-GATCTCGAGCTCAAGCTTCGATG**GACTACAAGGACGACGACGACAAG**GGGCCGCCTAAG-3′ and 5′-GGGCCGTCGACTGCAGAATTTTAGAAGCACTTGCGGTG-3′, with the coding sequence of the FLAG peptide DYKDDDDK shown in bold. pGEM-FLAG-NLS-beta-actin-S14C mutant was generated by site-directed mutagenesis using primers 5′-GTCGTCGACAACGGCTGCGGCATGTGCAAAGCC-3′ and 5′-GGCTTTGCACATGCCGCAGCCGTTGTCGACGAC-3′. pGEM-FKBP12^F36V^-mClover3 was generated by linearising pGEM-mClover3 using primers 5′-AATTCTGCAGTCGACGGC-3′ and 5′-CGAAGCTTGAGCTCGAGATC-3′, and transferring into it by Gibson assembly (lower case nucleotides indicate vector homology sequence) 5′-gatctcgagctcaagcttcgATGGGAGTGCAGGTGGAAAC-3′ and 5′-gggccgtcgactgcagaattGCCGCCTTCCAGTTTTAG-3′ flanked FKBP12^F36V^ synthetic coding sequence (Nabet et al., 2018). pGEM-FKBP12^F36V^-SNAP-NLS-beta-actin-S14C was generated by linearising pGEM with 5′-ATGGACAAAGACTGCGAAATG-3′ and 5′-TGAGCTCGAGATCTGAGAC-3′, and transferring into it by Gibson assembly 5′-ggtctcagatctcgagctcaATGGGAGTGCAGGTGGAAAC-3′ and 5′-atttcgcagtctttgtccatGCCGCCTTCCAGTTTTAG-3′ flanked FKBP12^F36V^. pGEM-FKBP12^F36V^-SNAP-NLS was generated by linearising pGEM with 5′-AATTCTGCAGTCGACGGC-3′ and 5′-CGAAGCTTGAGCTCGAGATC-3′, and transferring into it by Gibson assembly 5′-gatctcgagctcaagcttcgATGGGAGTGCAGGTGGAAAC-3′ and 5′-gggccgtcgactgcagaattTTACACCTTCCGCTTTTTCTTAGG-3′ flanked FKBP12^F36V^ from pGEM-FKBP12^F36V^-SNAP-NLS-beta-actin-S14C. pGEM-FKBP12^F36V^-FLAG-NLS was generated by linearising pGEM-FLAG-NLS with 5′-GGGCCGCCTAAGAAAAAG-3′ and 5′-CTTGTCGTCGTCGTCCTTG-3′, and transfer into it by Gibson assembly 5′-acaaggacgacgacgacaagATGGGAGTGCAGGTGGAAAC-3′ and 5′-cgctttttcttaggcggcccTTCCAGTTTTAGAAGCTCCACATC-3′ flanked FKBP12^F36V^. pGEM-FKBP12^F36V^-FLAG-NLS-beta-actin-S14C was generated by linearising pGEM-FLAG-NLS-beta-actin-S14C with 5′-GGGCCGCCTAAGAAAAAG-3′ and 5′-CTTGTCGTCGTCGTCCTTG-3′, and transferring into it by Gibson assembly 5′-acaaggacgacgacgacaagATGGGAGTGCAGGTGGAAAC-3′ and 5′-cgctttttcttaggcggcccTTCCAGTTTTAGAAGCTCCACATC-3′ flanked FKBP12^F36V^. pGEM-F-Tractin-NLS was generated by linearising pGEM-mClover3 with 5′-AATTCTGCAGTCGACGGC-3′ and 5′-CGAAGCTTGAGCTCGAGATC-3′, and transferring into it by Gibson assembly 5′-gatctcgagctcaagcttcgATGGCGCGGCCACGGGGC-3′ and 5′-gggccgtcgactgcagaattTGCCCTAGATCGCAAACCACTCACCTTCCG-3′ flanked synthetic coding sequence of mouse F-Tractin (70–199 bp in NM_146125.2). pGEM-mEGFP-UtrCH-NLS was generated by inserting a BamHI-NotI flanked synthetic coding sequence of UtrCH-NLS into the BamHI-NotI site of pGEM-mEGFP-C1 (Mogessie and Schuh, 2017).

Capped mRNA was synthesised using T7 polymerase (mMessage mMachine kit, following the manufacturer's instructions, Ambion). mRNA concentrations were determined by measurement on a Nanodrop spectrophotometer (Thermo Scientific).

### Confocal and super-resolution live imaging

Images were acquired with a Zeiss LSM800 microscope at 37°C. Oocytes were imaged in M2 medium under mineral oil using a 40× C-Apochromat 1.2 NA water-immersion objective as described in more detail by Mogessie (2020). Super-resolution time-lapse images were acquired using the Airyscan module on a Zeiss LSM800 microscope and processed post-acquisition using ZEN2 software (Zeiss).

### Immunofluorescence microscopy

We have recently published detailed protocols for optimised staining of actin filaments, which were used in this study to visualise nuclear F-actin structures (Mogessie, 2020). Mouse and sheep oocytes were fixed with 100 mM HEPES, 50 mM EGTA, 10 mM MgSO_4_, 2% formaldehyde (v/v), and 0.5% Triton X-100 (v/v) at 37°C for 25–30 min (mouse) or for 60 min after 10 s pre-permeabilisation in 0.4% Triton X-100 (v/v) in water (sheep). Oocytes were extracted in PBS supplemented with 0.3% Triton X-100 (v/v) at 4°C overnight. Antibody, F-actin and chromosome staining were performed for 2–2.5 h in PBS, 3% BSA (w/v), and 0.1% Triton X-100 (v/v) at room temperature. The nuclear envelope was stained by using primary rabbit anti-Lamin A/C antibody (ab133256, Abcam; 1:500) and Alexa-Fluor-647-labelled secondary anti-rat (Molecular Probes; 1:400) antibodies. F-actin was stained with Rhodamine or Alexa-Fluor-488 phalloidin (Molecular Probes; 1:20). DNA was stained with 5 µg/ml Hoechst dye 33342 (Molecular Probes).

Confocal and Airyscan super-resolution images were acquired with a Zeiss LSM800 confocal microscope equipped with a 40× C-Apochromat 1.2 NA water-immersion objective. Images under control and perturbation conditions were acquired by using identical imaging conditions.

### Drug addition and fluorescence labelling experiments

To disrupt actin, oocytes were treated for 1 h with Cytochalasin D (C8273-1MG, Merck) at a final concentration of 5 μg/ml, Latrunculin B (428020-1MG, Merck) at a final concentration of 5 μM or CK-666 (182515-25MG, Merck) at a final concentration of 200 μM in M2 medium supplemented with dbcAMP. To block nuclear import, oocytes were treated with a combination of 100 μM Importazole (SML0341-5MG, Merck) and 30 μM Ivermectin (I8000010, Merck). We have found that a combination of these two drugs is a much tighter block of nuclear import in mouse oocytes. This combination was thus used to achieve the maximal effect of inhibition in our analyses. These final concentrations were maintained in experiments where oocytes were simultaneously treated with Cytochalasin D, Importazole and Ivermectin. For rapid protein degradation experiments, prophase-arrested oocytes were treated with and matured in 100 nM dTAG-13 (SML2601, Merck). All drugs were dissolved in DMSO (D2650-5X5ML, Merck). Where DMSO was used as control, it was diluted in M2 medium supplemented with dbcAMP, identically to corresponding experimental conditions. SNAP-tagged proteins expressed in oocytes from microinjected mRNA were fluorescence labelled by incubating cells with 3 μM SNAP-Cell 647-SiR (S9102S, NEB) in M2 medium for 30 min and for a further 30 min in M2 medium without substrate.

To fluorescence label nuclear actin structures, nuclear actin chromobody was expressed in oocytes at variable concentrations. For low-level expression, oocytes were microinjected with 6–8 pl of 0.02 μg/μl nuclear actin chromobody mRNA followed by expression for 1–3 h. For high-level expression, oocytes were microinjected with 6–8 pl of 0.2 μg/μl nuclear actin chromobody mRNA followed by expression for 2 h.

### Chromosome alignment and segregation analysis

For chromosome alignment and segregation analyses, images were acquired at a temporal resolution of 5 min and with a *Z*-stack thickness of ∼40 µm at 1.5 µm confocal sections. Chromosomes that were distinctly separate from the metaphase plate chromosome mass at the time of anaphase onset (shown in Fig. 4D) were scored as misaligned chromosomes. Chromosomes that fell behind the main mass of segregating chromosomes for at least 10 min after anaphase onset were scored as lagging chromosomes. For both quantifications, maximum intensity projections of only those metaphase spindles that were oriented parallel to the imaging plane at and during anaphase were analysed.

### Isosurface reconstruction and 3D volume quantification of nuclear actin filaments

For 3D volume quantification of nuclear actin filaments, confocal images of nuclei in fixed oocytes were typically acquired at a spatial resolution of 1 μm confocal sections covering 45 μm. Isosurfaces, i.e. 3D surface representations of points with equal values in a 3D data distribution, corresponding to nuclear membranes were reconstructed in 3D by using the Cell module of Imaris software (Bitplane) and the immunofluorescence signal of nuclear envelope antibodies. The nuclear isosurface was used to mask F-actin signal and to remove cytoplasmic F-actin structures surrounding the nuclei. In the masked region, 3D isosurfaces of fluorescent phalloidin-labelled nuclear actin filaments were reconstructed using similar settings between different experimental groups within each repetition. Individual values regarding the volume of nuclear F-actin were normalised to the mean value of the control group for graphical presentation.

### Fluorescence intensity quantification of cytoplasmic F-actin

To quantify the density of the cytoplasmic actin network, single-section super-resolution Airyscan images of fluorescent phalloidin-labelled cytoplasmic actin filaments were acquired. Measured in the cytoplasm was the mean fluorescence intensity of actin filaments of six to twelve regions per oocyte; this was then averaged to generate cytoplasmic F-actin intensity values for each oocyte. Background subtraction was performed in ImageJ by subtracting the mean fluorescence intensity value of a region outside each oocyte from its corresponding cytoplasmic F-actin intensity value. For graphical presentation, individual fluorescence intensity values were normalised to the mean intensity value of the control group.

### Four-dimensional tracking of prophase oocyte chromatin movement

For analyses of chromatin mobility, nuclear membranes in prophase-arrested oocytes were labelled by microinjecting and expressing fluorescent lamin nanobodies (lamin chromobody). In genetic nuclear actin stabilisation experiments, FLAG-NLS or FLAG-NLS-beta-actin-S14C mRNA was co-expressed with nuclear lamin nanobodies. To label DNA, oocytes were incubated with 250 nM 5-SiR-Hoechst (Bucevicius et al., 2019) in DMSO for 2 h before the imaging experiment but during mRNA expression. In chemical nuclear actin stabilisation experiments, oocytes were incubated in DMSO or Cytochalasin D 1 h before the imaging experiment but during mRNA expression. Confocal images of the nuclear envelope and chromosomes were acquired at a temporal resolution of 100 s; *Z*-stack thickness was 36 μm and thickness of confocal sections was 1.5 µm.

To exclude the contribution of nuclear movements to chromatin mobility, isosurfaces of the nuclear envelope were reconstructed by using the Cell module of Imaris software (Bitplane) and the fluorescent nuclear chromobody signal. Four-dimensional (4D; *x*, *y*, *z*, time) nuclear movement tracks obtained from these reconstructions were then used to automatically correct translational and rotational drift in Imaris. Isosurfaces of prominent chromatin spots were next reconstructed in 3D by using the 5-SiR-Hoechst fluorescence signal and automatically tracked to generate 4D tracks that were manually corrected to remove inaccurate trajectories. Five to eight tracks of separate chromatin masses per oocyte were obtained and analysed through this pipeline.

### Statistical data analyses

Histograms, statistical box plots and other graphs were generated using OriginPro software (OriginLab). Statistical box plots represent median (line), mean (small square), 5th, 95th (whiskers) and 25th and 75th percentile (box enclosing 50% of the data) and are overlaid with individual data points. Average (mean), standard deviation and statistical significance based on two-tailed Student's *t*-test or Fisher's exact test were calculated in OriginPro software (OriginLab). All error bars represent standard deviations. Two-way analysis of variance was performed in Prism software (GraphPad). Significance values are *P<0.05, **P<0.005 and ***P<0.0005. Non-significant values are indicated as N.S.

## Supplementary Material

Supplementary information
