# Supplementary material for: The prophase oocyte nucleus is a homeostatic G-actin buffer
Source: J Cell Sci. 2022 Mar 23;135(6):jcs259807. doi: 10.1242/jcs.259807 (PMC8977058; doi:10.1242/jcs.259807)
Supplement: Supplementary information [file joces-135-259807-s1.pdf]

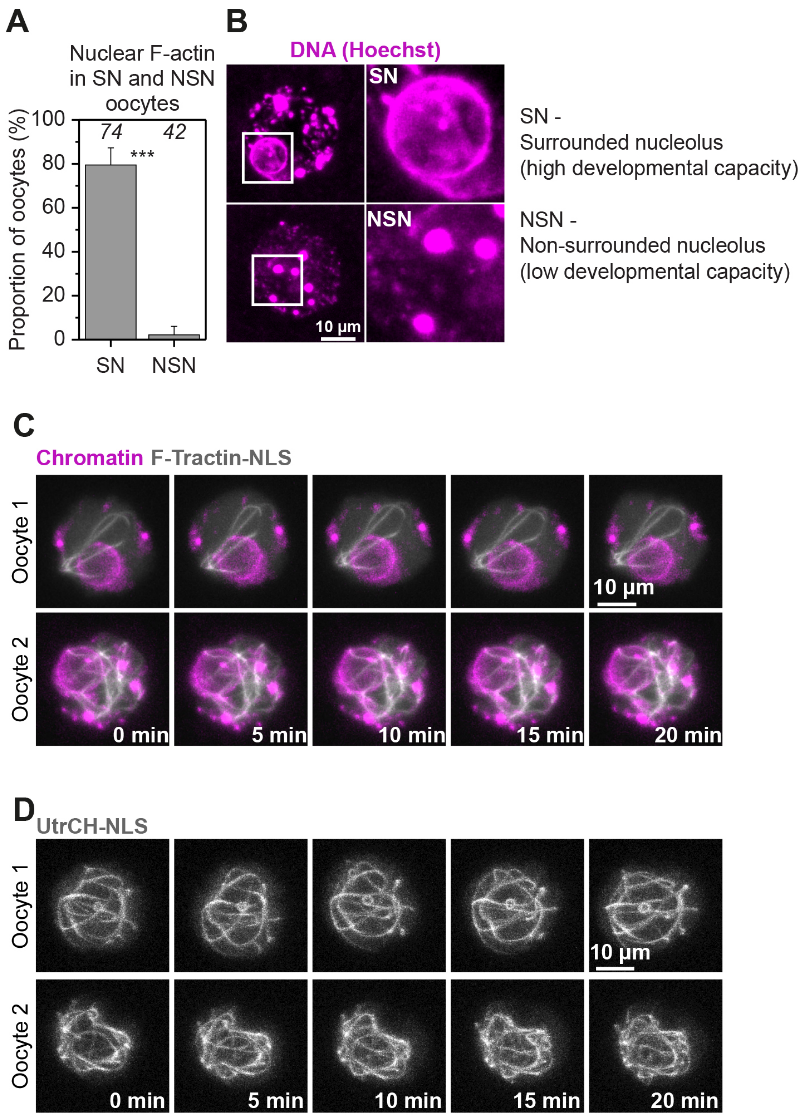

Journal of Cell Science • Supplementary information

Fig. S1 Nuclear F-actin is a common feature in healthy mammalian oocytes

(**A**) Quantification of nuclear F-actin presence in prophase-arrested mouse oocytes with surrounded nucleolar (SN) and non-surrounded nucleolar (NSN) chromatin configuration. Data are from 3 independent experiments. Fisher's exact test was used to test for significance.

(**B**) Representative images of surrounded nucleolar (SN) and non-surrounded nucleolar (NSN) chromatin (magenta) configuration in prophase-arrested oocytes. Boxes mark regions that are magnified in insets.

(**C**) Panels from time lapse movies of nuclear F-actin in two prophase-arrested mouse oocytes expressing mClover3-F-Tractin-NLS and H2B-mRFP (to label chromatin).

(**D**) Panels from time lapse movies of nuclear F-actin two prophase-arrested mouse oocytes expressing mEGFP-UtrCH-NLS and H2B-mRFP.

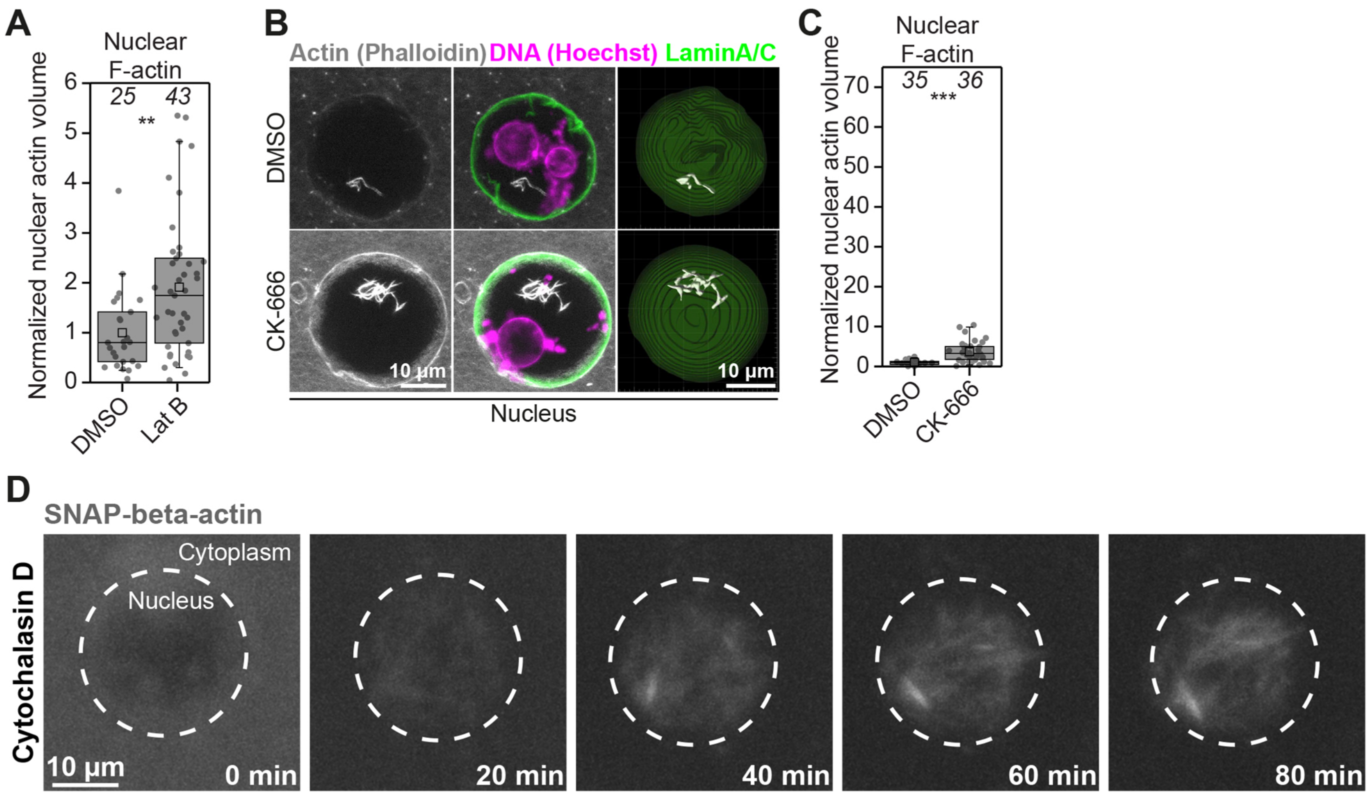

Journal of Cell Science • Supplementary information

Fig. S2 Excess cytosolic G-actin causes uncontrolled nuclear F-actin assembly.

(A) Quantification of nuclear F-actin volumes from isosurface reconstructions in 2D in DMSO- or Latrunculin B-treated mouse oocytes. Data are from 3 independent experiments. Y-axis scaling is adjusted to show higher resolution distribution of data shown in Fig. 2F.

(B) Maximum intensity projections (9 confocal sections) of Phalloidin labelled nuclear actin filaments (grey), DNA (magenta) and nuclear membrane (green) in DMSO- or CK-666-treated mouse oocytes.

(C) Quantification of nuclear F-actin volumes from isosurface reconstructions in B in DMSO- or CK-666-treated mouse oocytes. Data are from 3 independent experiments.

(D) Panels from time lapse movies of nuclear F-actin assembly in a Cytochalasin D-treated prophase-arrested mouse oocyte expressing SNAP-beta-actin (labelled with SNAP-Cell 647-SiR). T =0 min denotes the start time of imaging immediately after drug addition..

Two-tailed Student’s t test was used to test for significance in A and C.

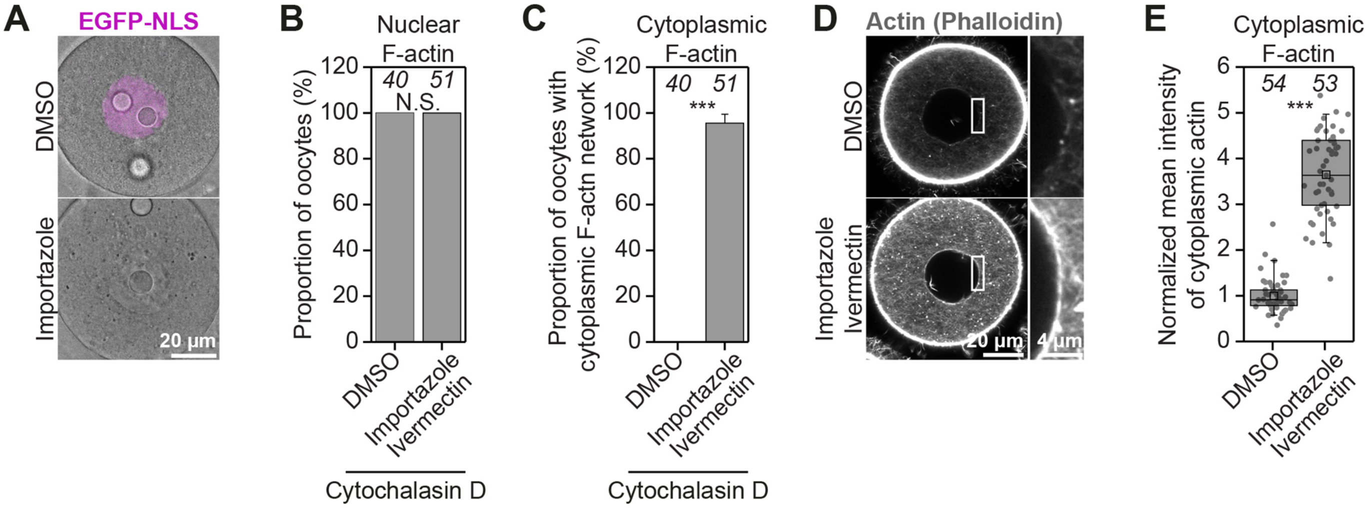

Fig. S3 Actin monomers are nuclear import cargoes in mouse oocytes.

(A) Representative images of GFP-NLS in DMSO- or Importazole-treated mouse oocytes.

(B) Quantification of nuclear F-actin presence in DMSO- or Importazole/Ivermectin-treated mouse oocytes that were then treated with Cytochalasin D. Data are from 3 independent experiments.

(C) Quantification of cytoplasmic F-actin network presence in DMSO- or Importazole/Ivermectin-treated mouse oocytes that were then treated with Cytochalasin D. Data are from 3 independent experiments.

(D) Single section Airyscan images of Phalloidin labelled cytoplasmic F-actin in DMSO- or Importazole/Ivermectin-treated mouse oocytes. Boxes mark regions that are magnified in insets.

(E) Quantification of cytoplasmic F-actin network fluorescence intensity in DMSO- or Importazole/Ivermectin-treated mouse oocytes. Data are from 3 independent experiments.
Statistical significance was tested using Fisher's exact test [(B) and (C)] and Two-tailed Student's t test (E).

Journal of Cell Science • Supplementary information

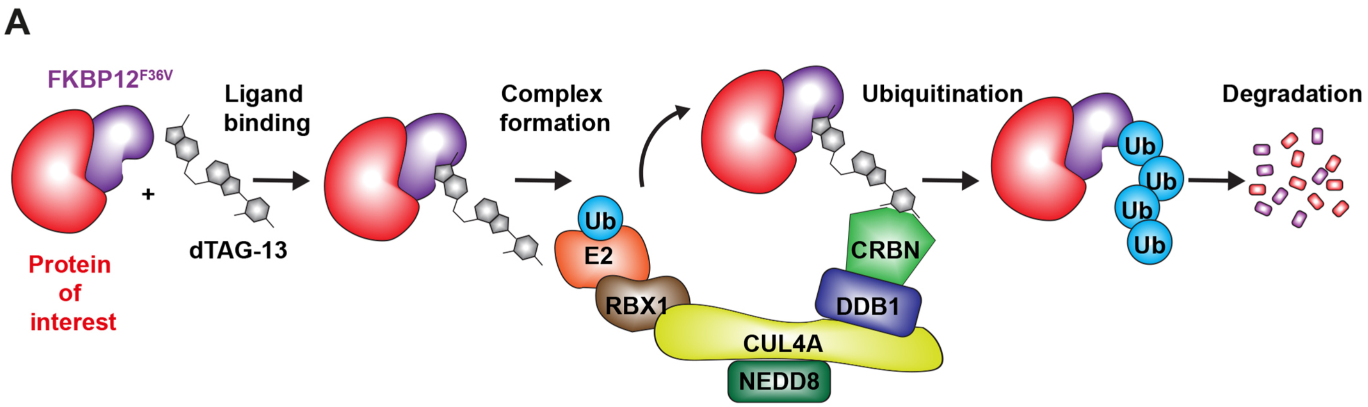

Fig. S4 Molecular strategy of dTAG-mediated targeted protein degradation.

(**A**) The binding of dTAG-13 molecules to FKBP12^{F36V} induces dimerization of FKBP12^{F36V} fusion proteins and the CRBN E3 ligase complex, thereby causing CRBN-mediated degradation of fusion proteins.

Journal of Cell Science • Supplementary information

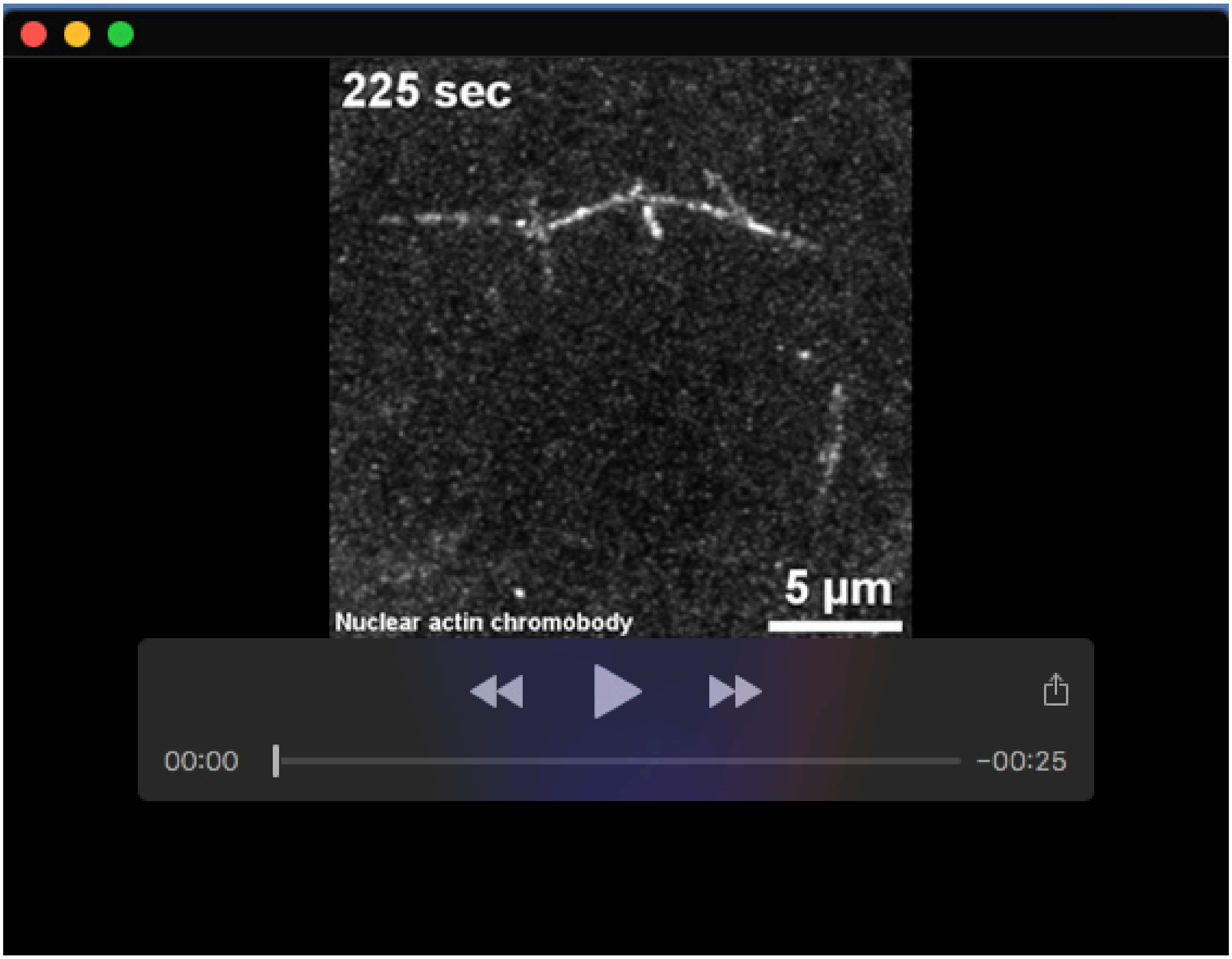

Movie 1. Time lapse movie of nuclear F-actin (labelled with low expression nuclear actin chromobody) in prophase-arrested mouse oocyte (oocyte 1)

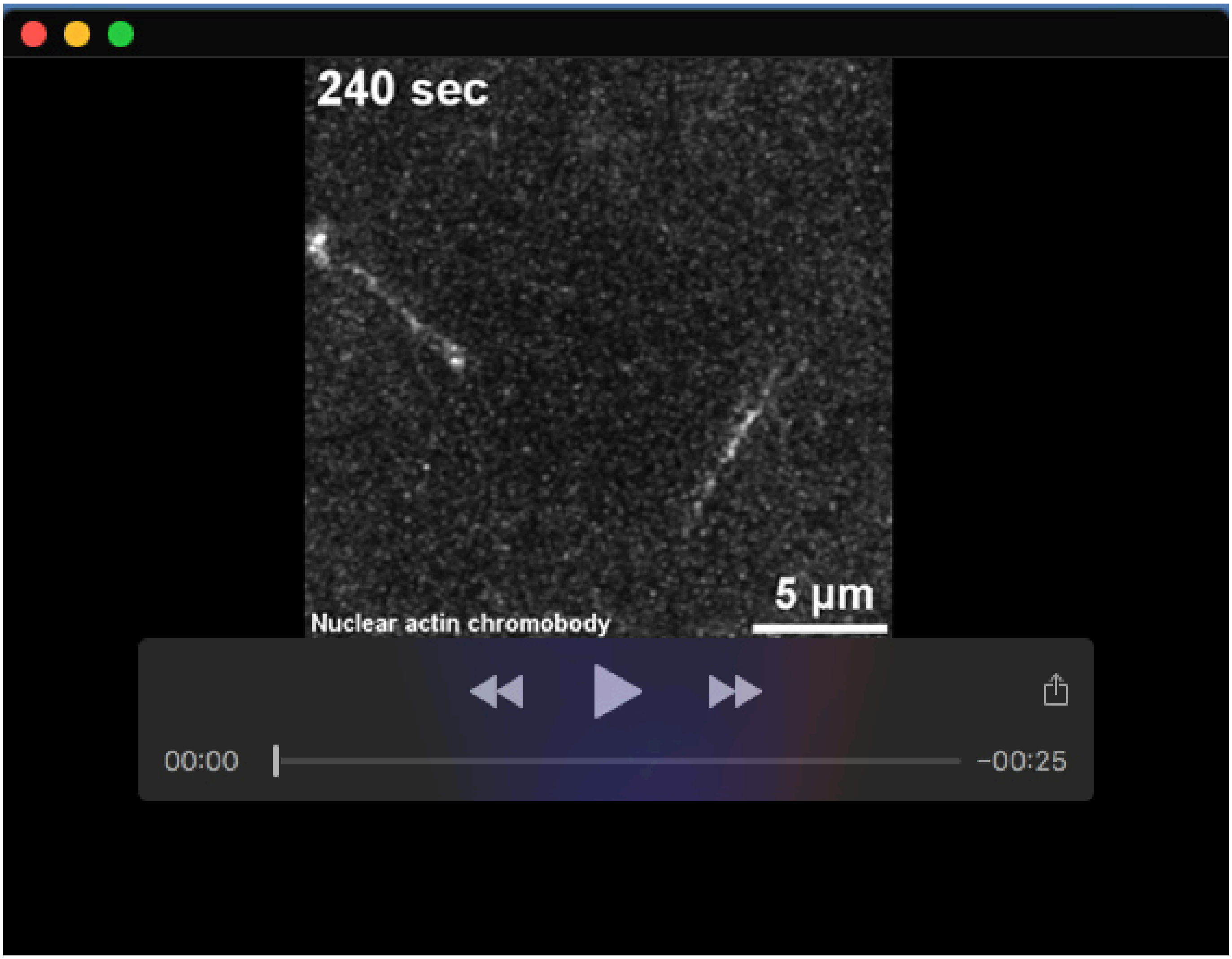

Movie 2. Time lapse movie of nuclear F-actin (labelled with low expression nuclear actin chromobody) in prophase-arrested mouse oocyte (oocyte 2)

Journal of Cell Science • Supplementary information

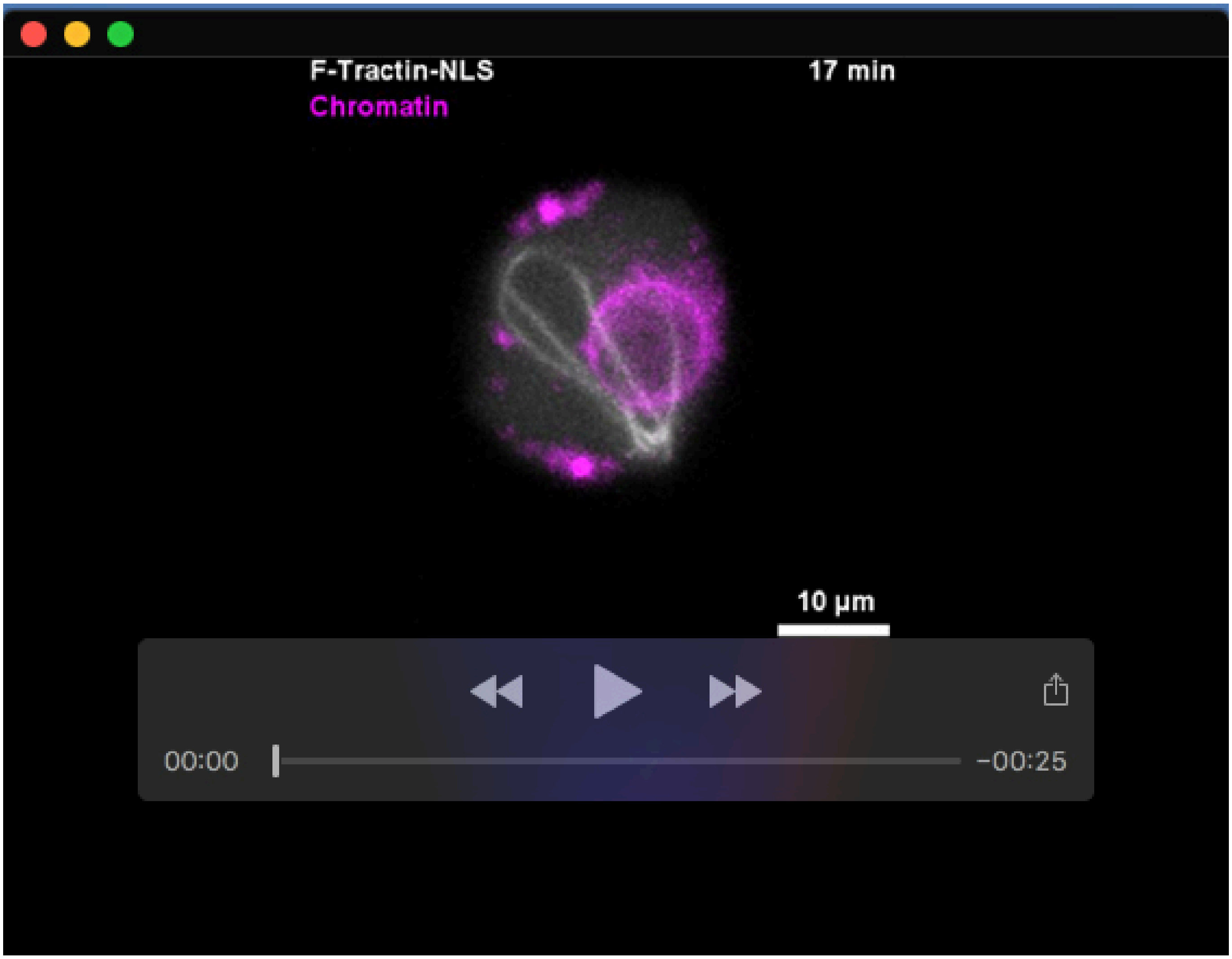

Movie 3. Time lapse movie of nuclear F-actin (labelled with F-tractin) and chromatin labelled with H2b-mRFP in prophase-arrested mouse oocyte (oocyte 1).

Journal of Cell Science • Supplementary information

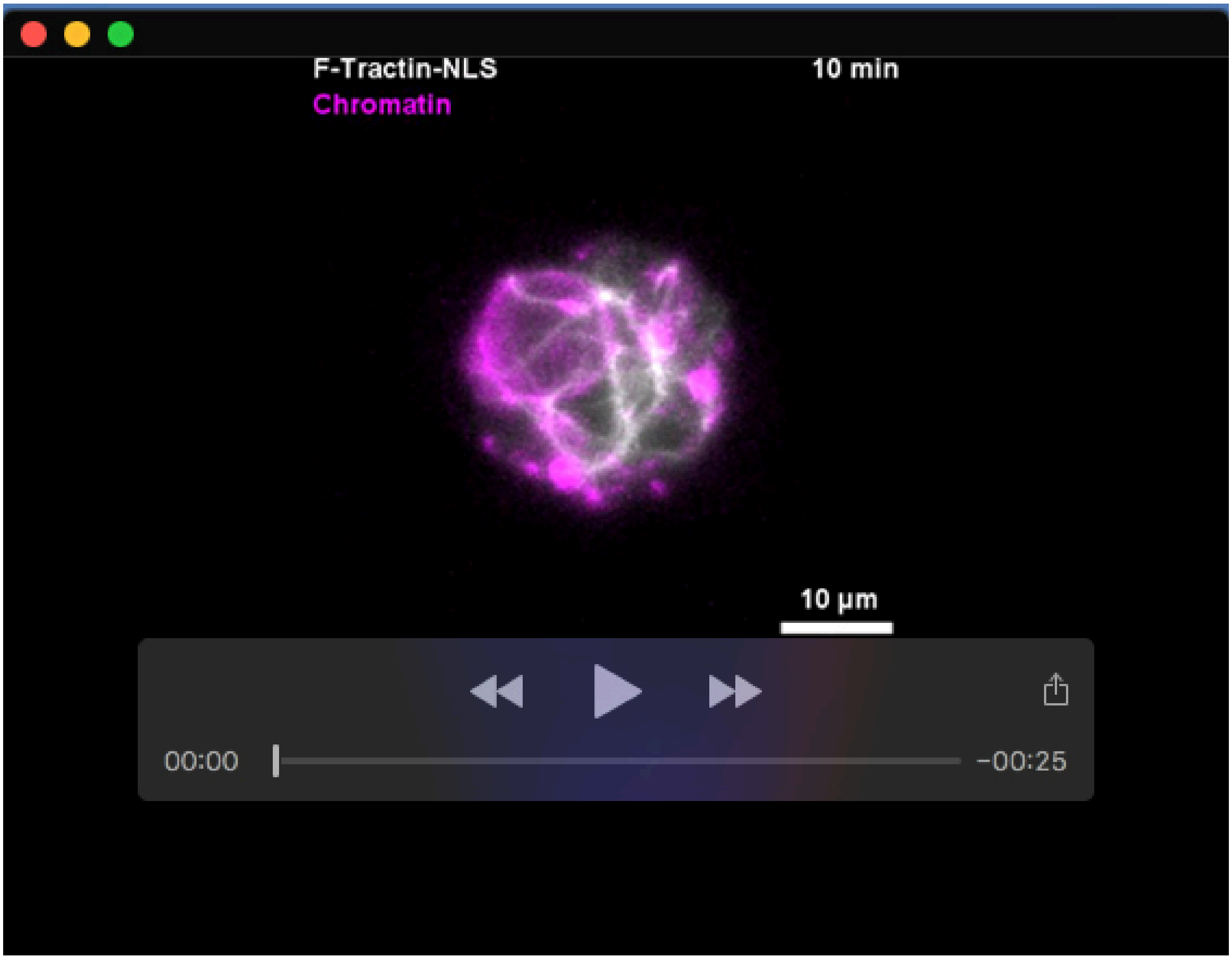

Movie 4 . Time lapse movie of nuclear F-actin (labelled with F-tractin) and chromatin labelled with 5-SiR-Hoechst in prophase-arrested mouse oocyte (oocyte 2).

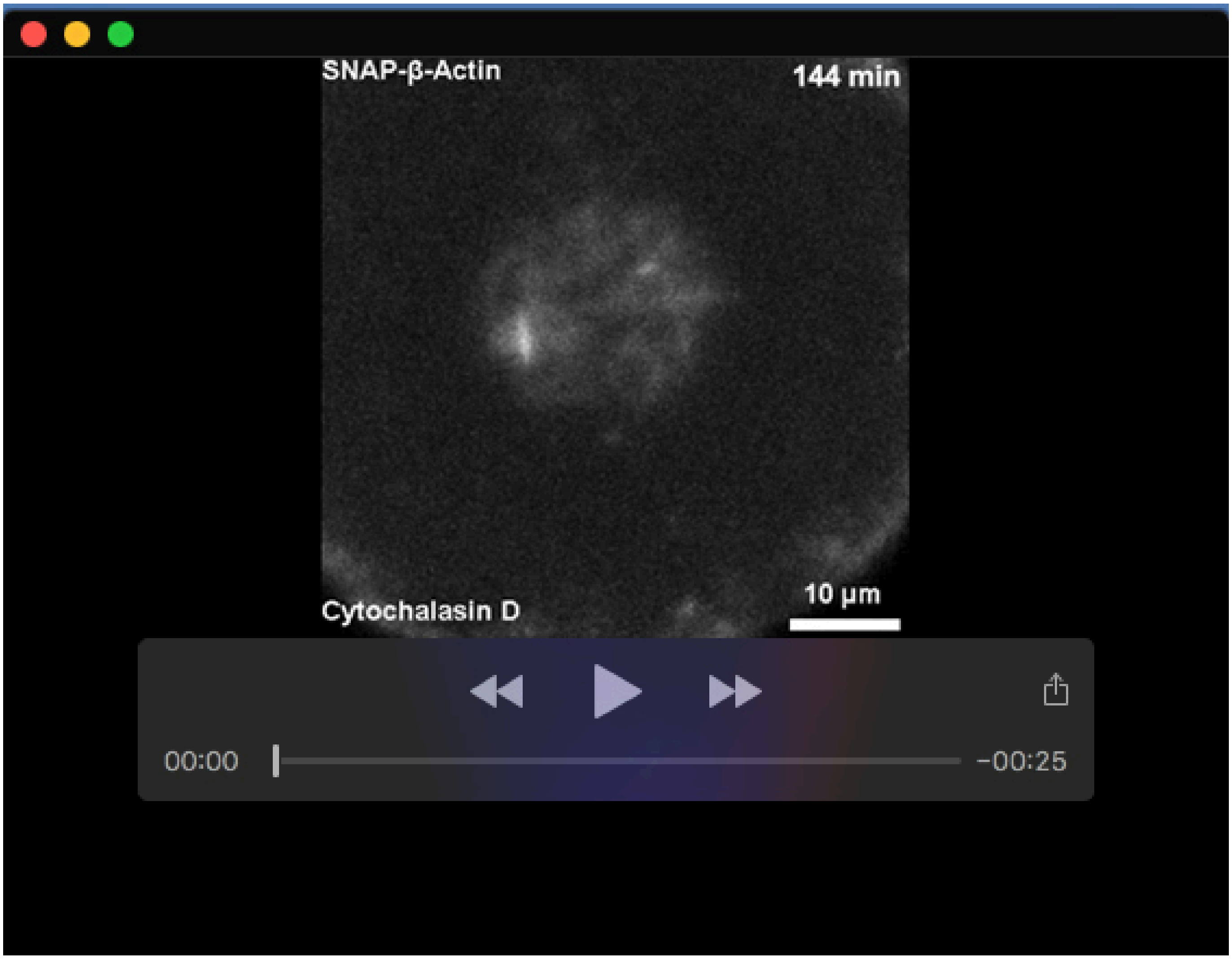

Movie 5. Time lapse movie of SNAP-beta-actin in a Cytochalasin D treated prophase-arrested mouse oocyte. T=0 denotes the start of imaging experiment immediately after drug addition.

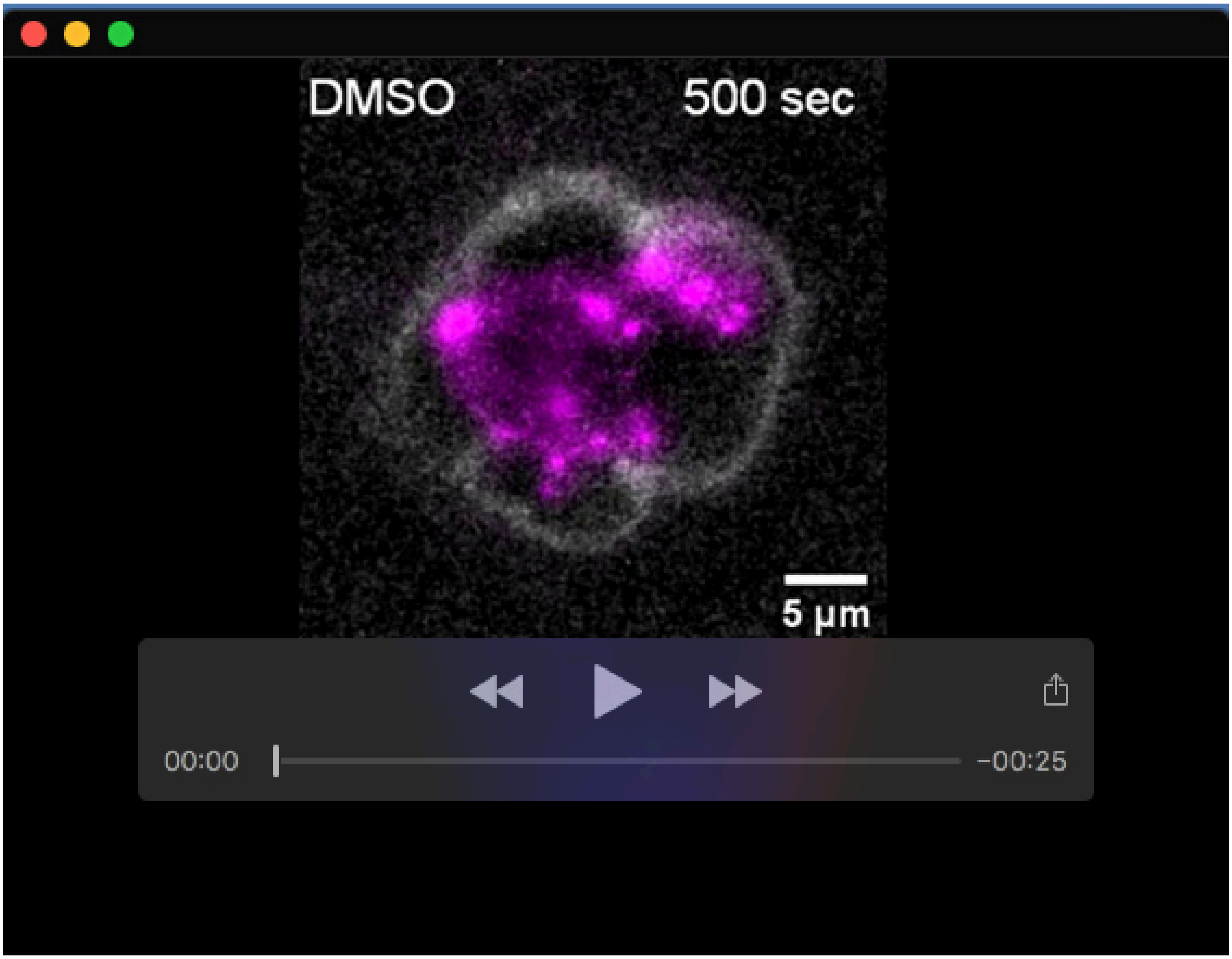

Movie 6. Time lapse movie of chromatin movement in a DMSO-treated mouse oocyte. Chromatin (magenta) is labelled with 5-SiR-Hoechst and nuclear membrane (grey) is labelled with lamin chromobody.

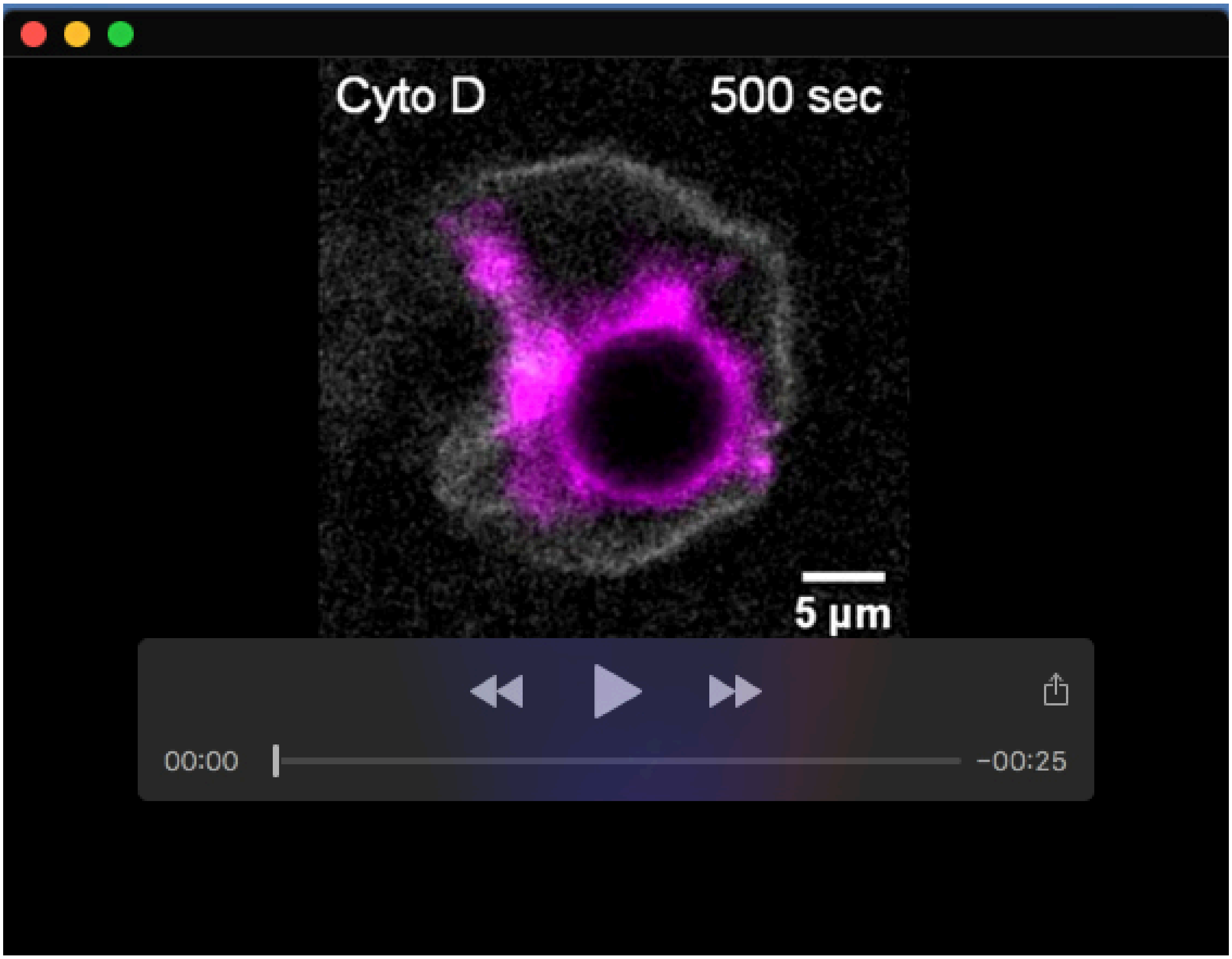

Movie 7. Time lapse movie of chromatin movement in a Cytochalasin D-treated mouse oocyte. Chromatin (magenta) is labelled with 5-SiR-Hoechst and nuclear membrane (grey) is labelled with lamin chromobody.

Journal of Cell Science • Supplementary information

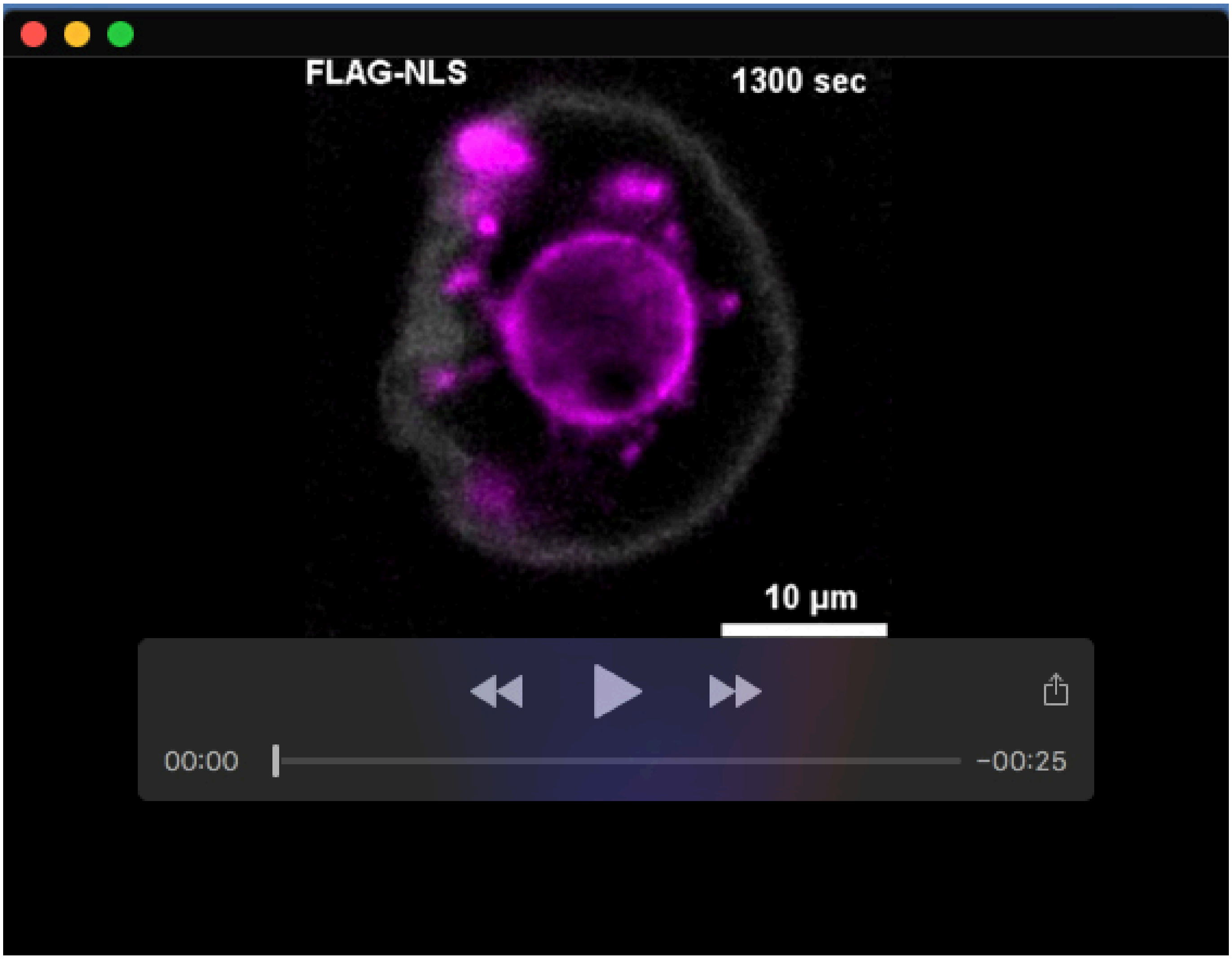

Movie 8. Time lapse movie of chromatin movement in a FLAG-NLS expressing control mouse oocyte. Chromatin (magenta) is labelled with 5-SiR-Hoechst and nuclear membrane (grey) is labelled with lamin chromobody.

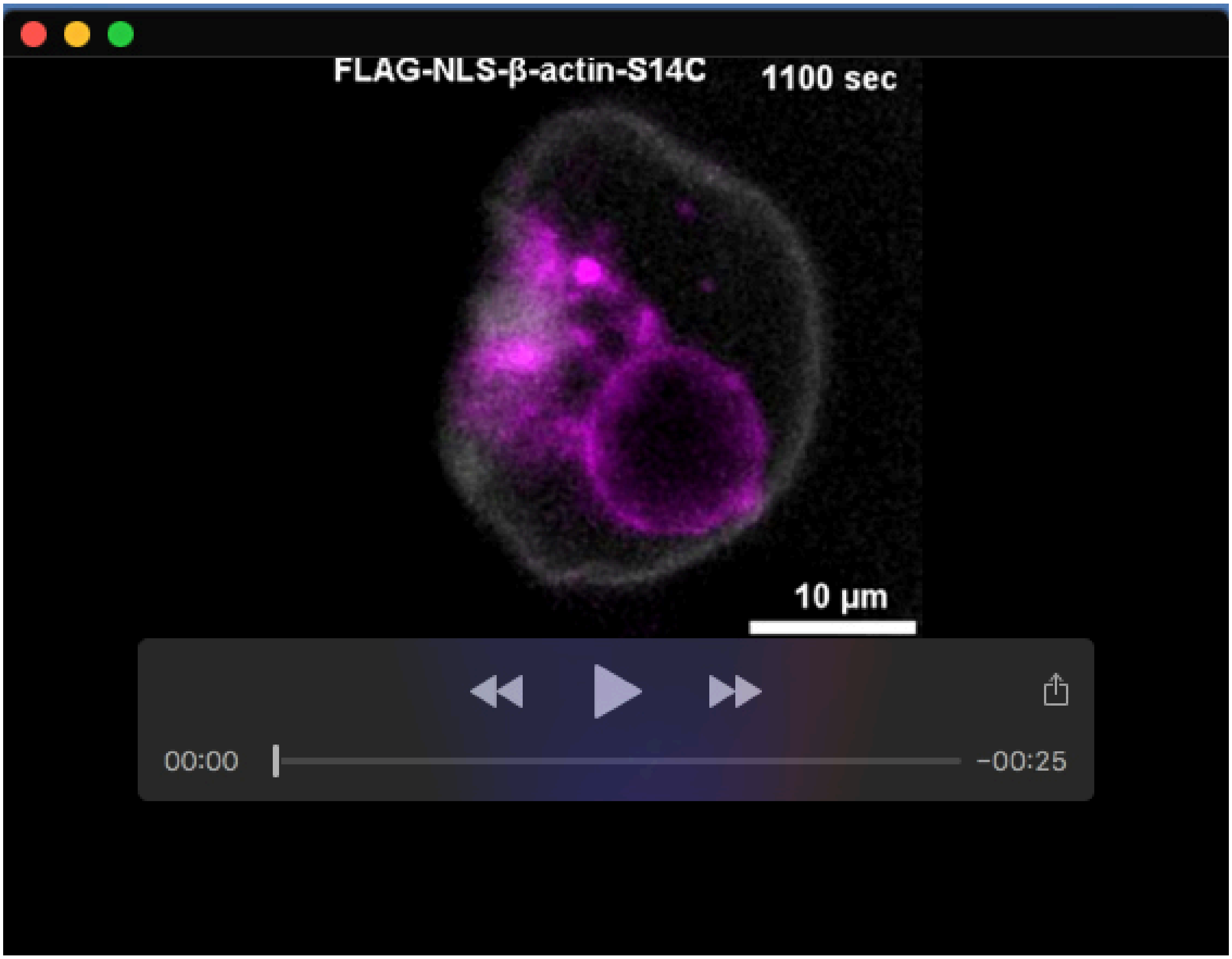

Movie 9. Time lapse movie of chromatin movement in a FLAG-NLS-beta-actin-S14C expressing mouse oocyte. Chromatin (magenta) is labelled with 5-SiR-Hoechst and nuclear membrane (grey) is labelled with lamin chromobody.

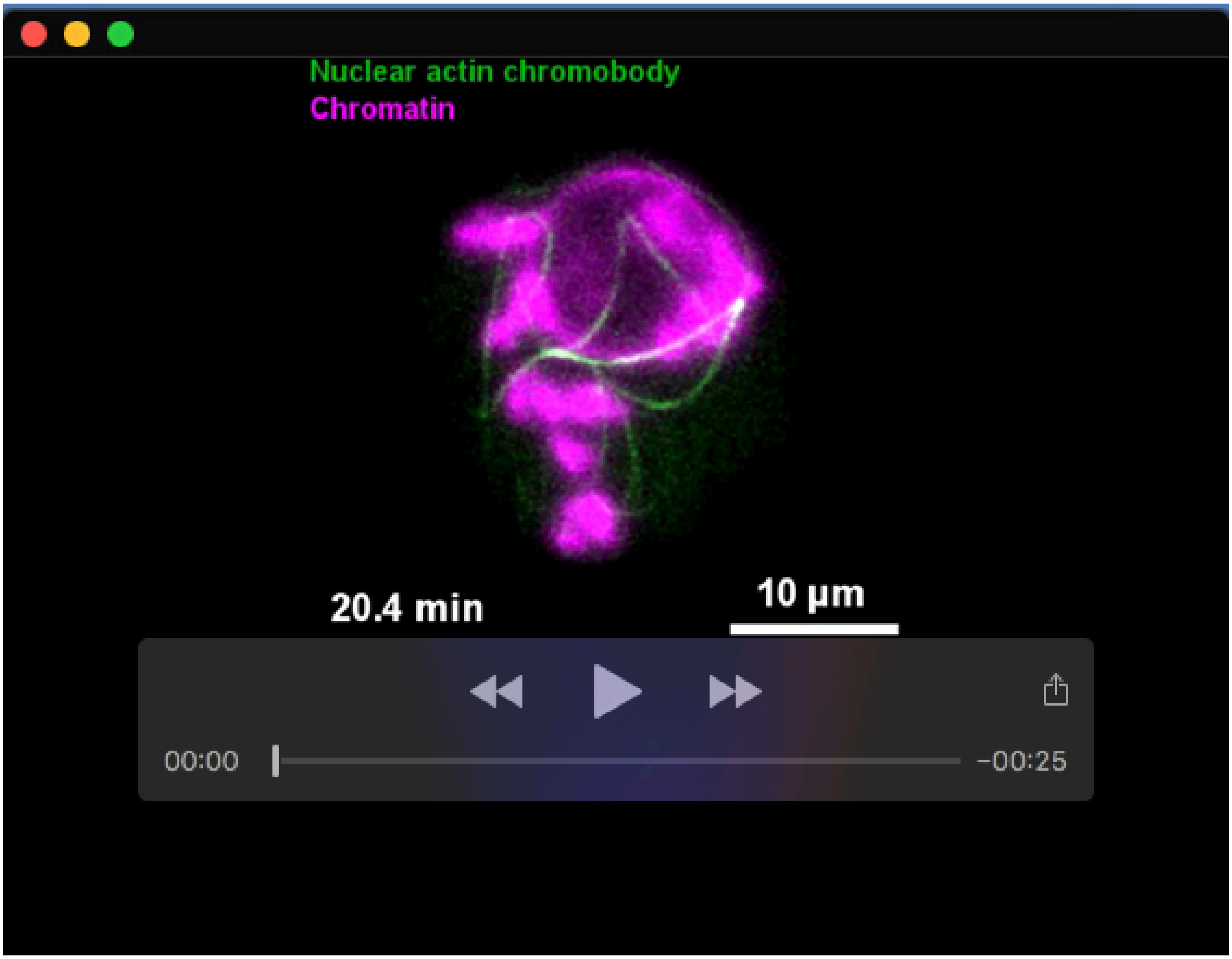

Movie 10. Time lapse movie of chromatin movement inside a mouse oocyte nucleus containing excess nuclear F-actin (induced by nuclear actin chromobody overexpression). Chromatin (magenta) is labelled with 5-SiR-Hoechst and nuclear F-actin (green) is labelled with nuclear actin chromobody.

Journal of Cell Science • Supplementary information

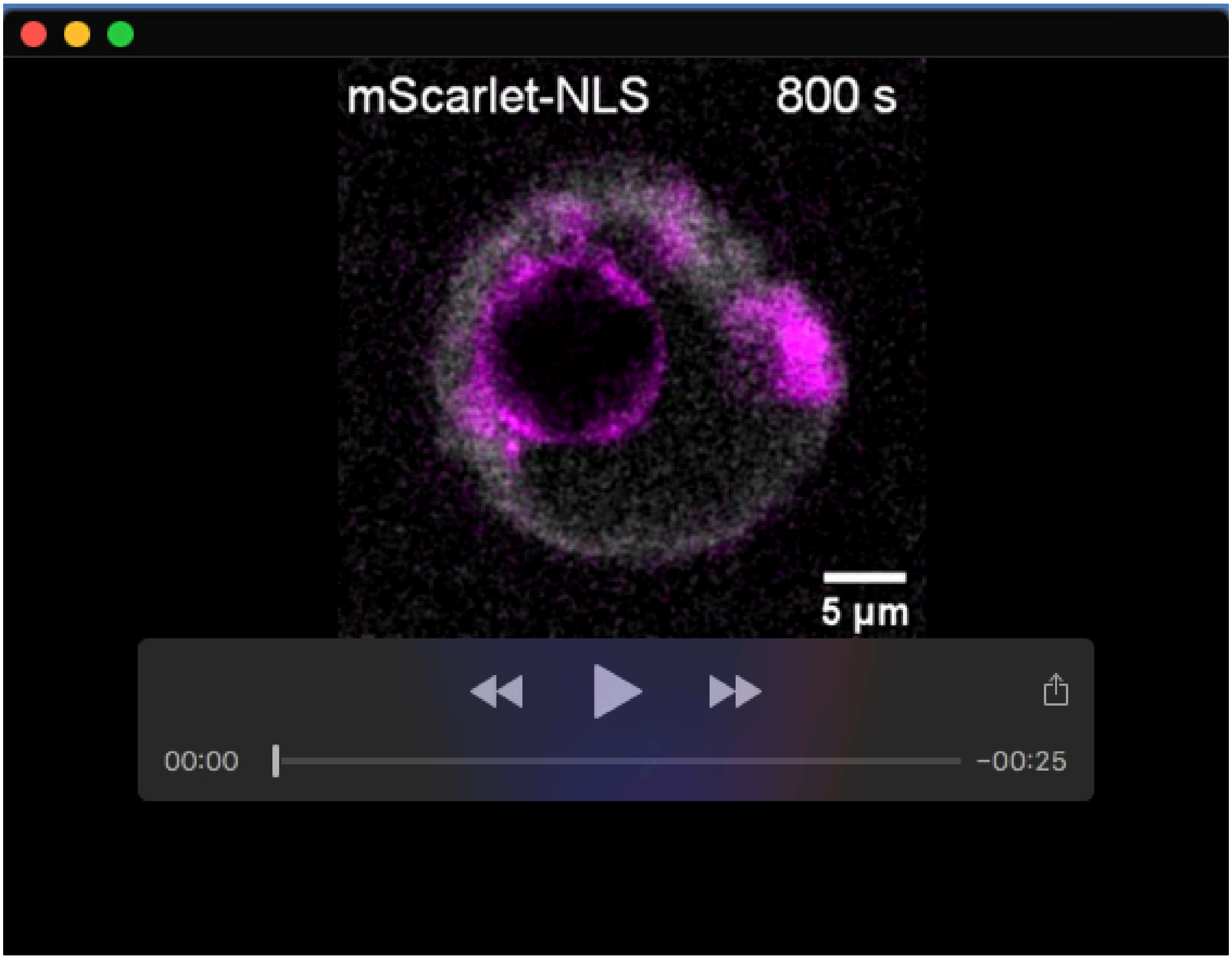

Movie 11. Time lapse movie of chromatin movement in a mScarlet-NLS expressing control mouse oocyte. Chromatin (magenta) is labelled with 5-SiR-Hoechst and nuclear membrane (grey) is labelled with lamin chromobody.

Journal of Cell Science • Supplementary information

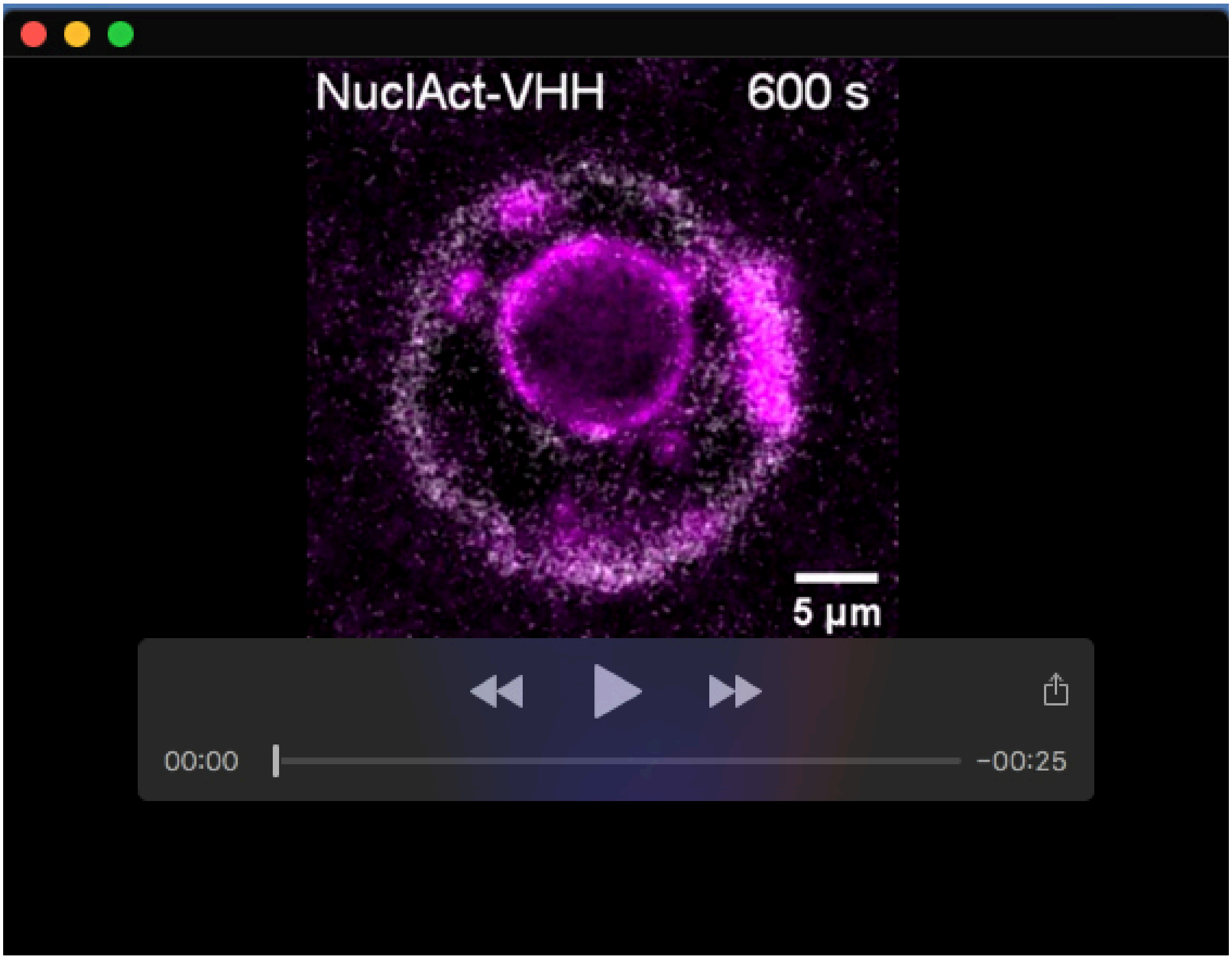

Movie 12. Time lapse movie of chromatin movement in a nuclear actin chromobody (NucAc-VHH) overexpressing mouse oocyte. Chromatin (magenta) is labelled with 5-SiR-Hoechst and nuclear membrane (grey) is labelled with lamin chromobody.

Journal of Cell Science • Supplementary information

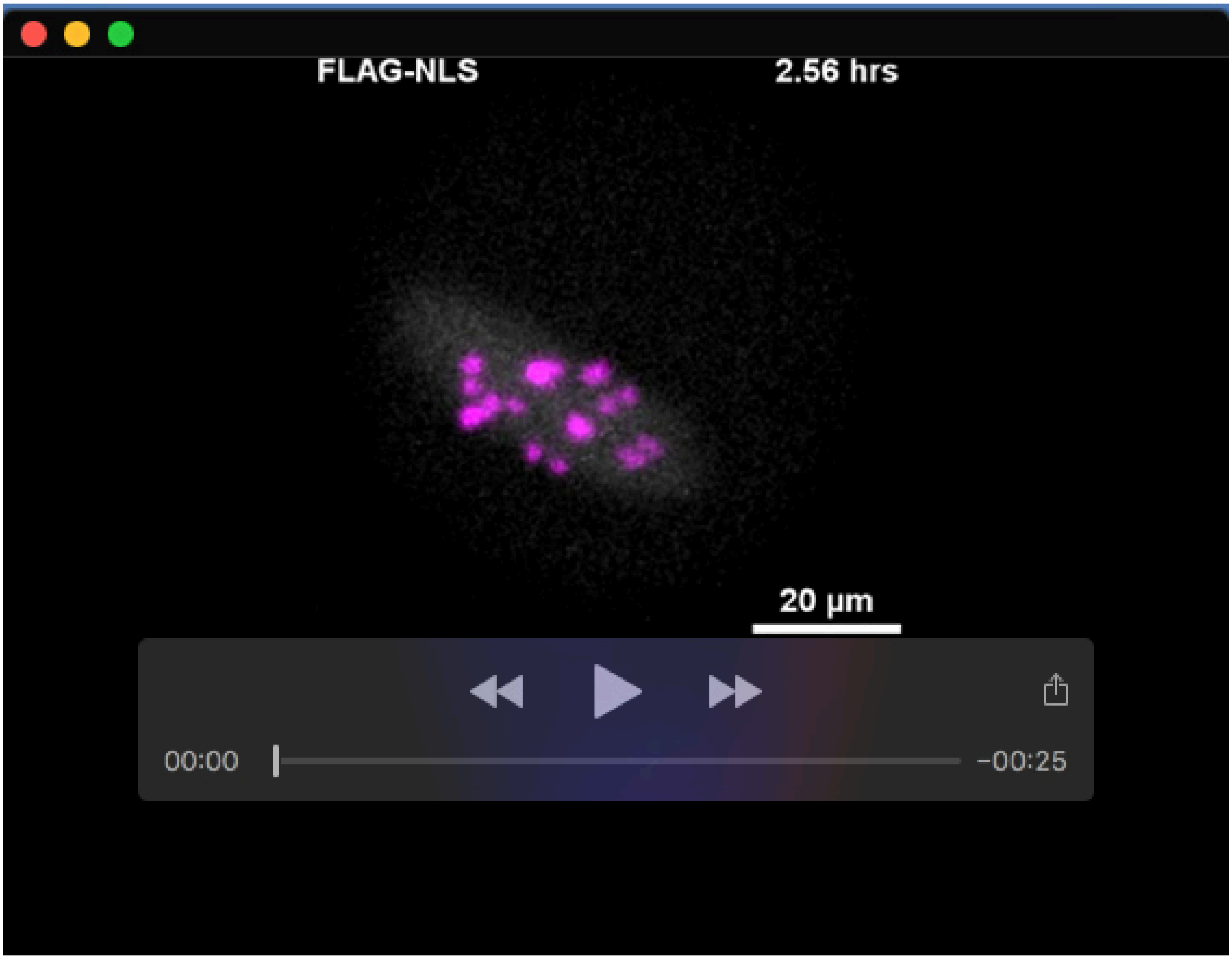

Movie 13. Time lapse movie of chromosome alignment and segregation during meiosis I in a FLAG-NLS expressing control mouse oocyte. Microtubules (grey) are labelled with EGFP-MAP4-MTBD and chromosomes (magenta) are labelled with H2B-mRFP.

Journal of Cell Science • Supplementary information

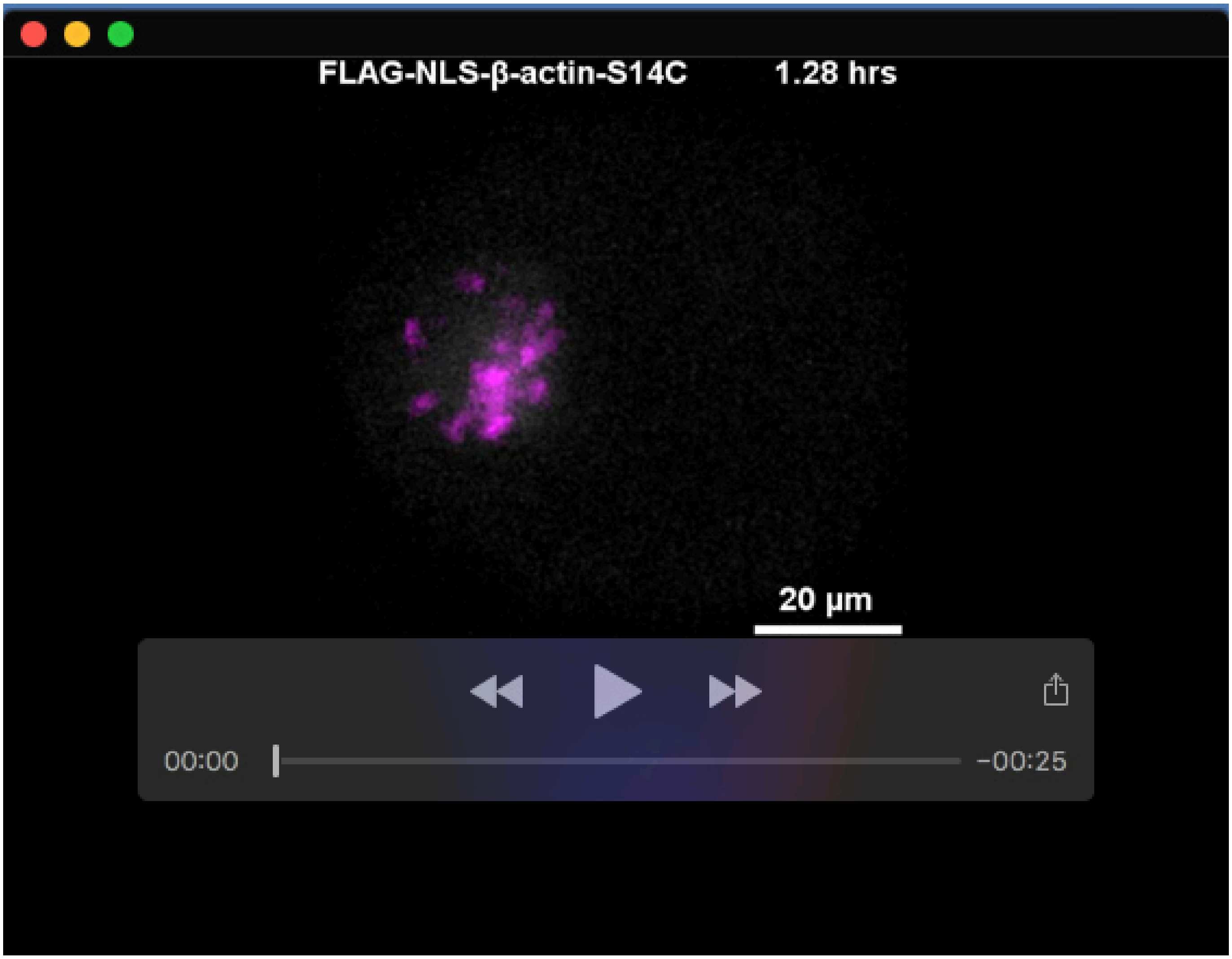

Movie 14. Time lapse movie of chromosome alignment and segregation during meiosis I in a FLAG-NLS-beta-actin-S14C expressing mouse oocyte. Microtubules (grey) are labelled with EGFP-MAP4-MTBD and chromosomes (magenta) are labelled with H2B-mRFP.

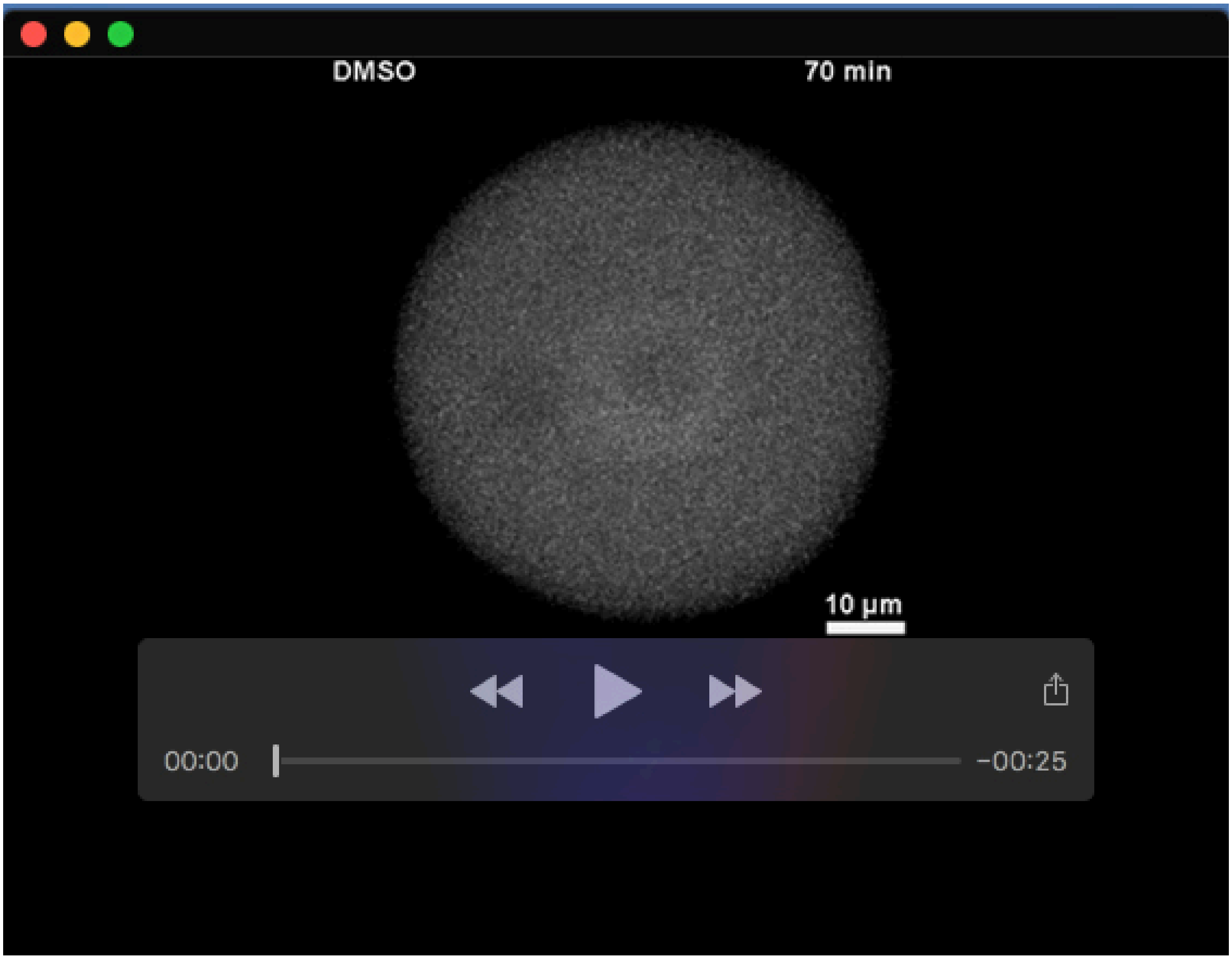

Movie 15. Time lapse movie of cytoplasmic fluorescence in a FKBP12^{F36V}-mClover3 expressing DMSO-treated control mouse oocyte.

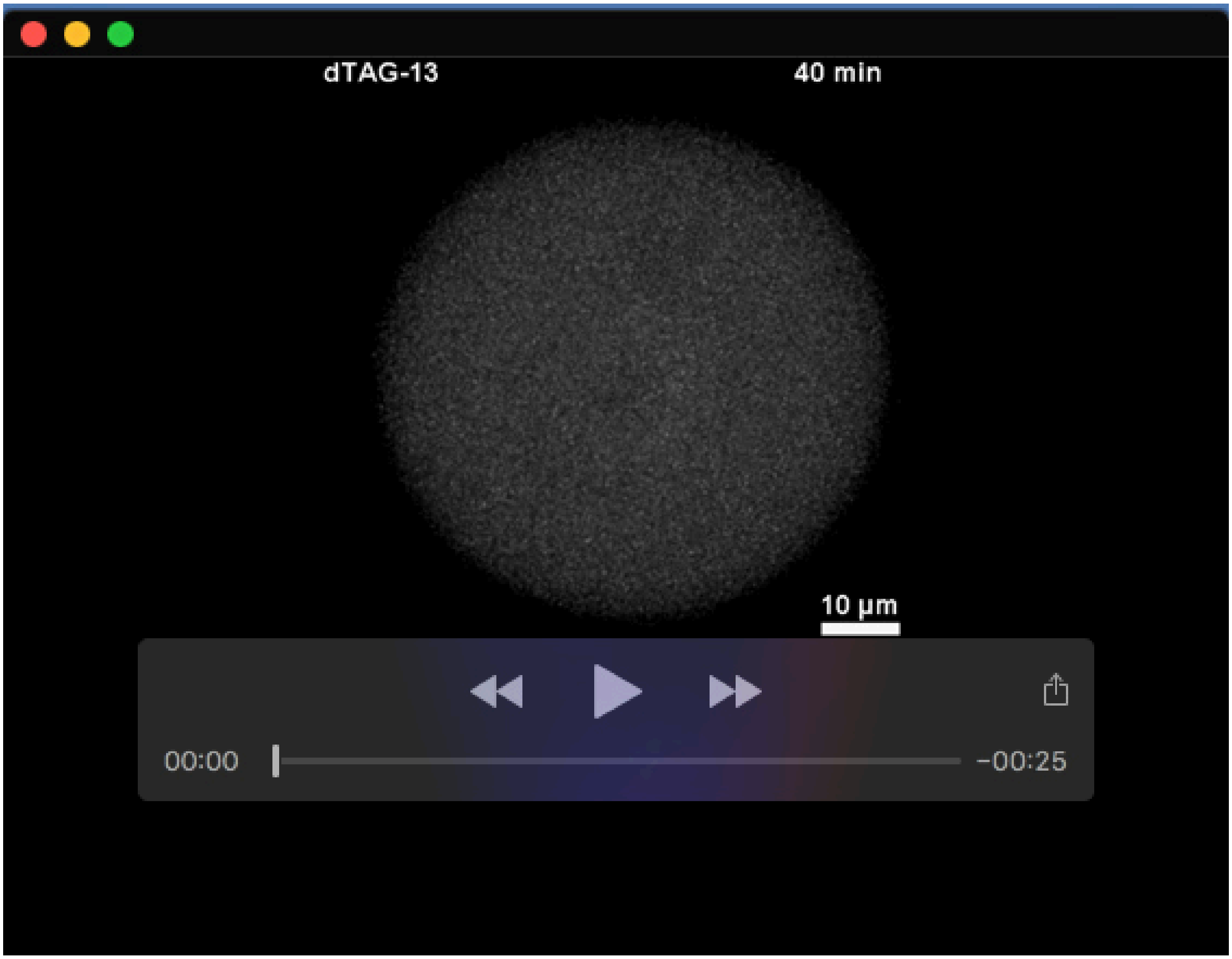

Movie 16. Time lapse movie of cytoplasmic fluorescence in a FKBP12^{F36V}-mClover3 expressing dTAG-13-treated control mouse oocyte.

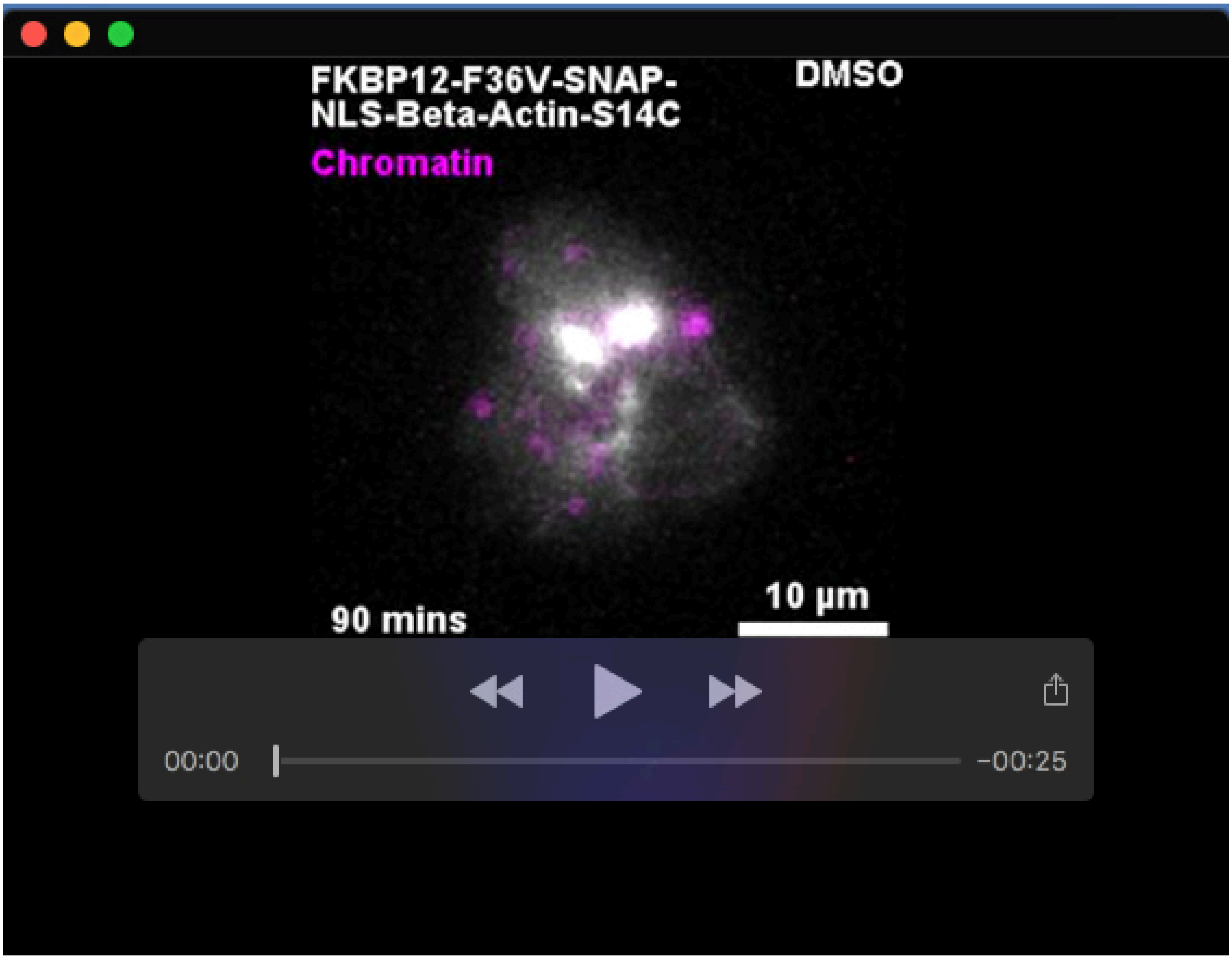

Movie 17. Time lapse movie of nuclear F-actin structures in FKBP12^{F36V}-SNAP-NLS-beta-actin-S14C expressing DMSO-treated control mouse oocyte. Chromatin (magenta) is labelled with 5-SiR-Hoechst.

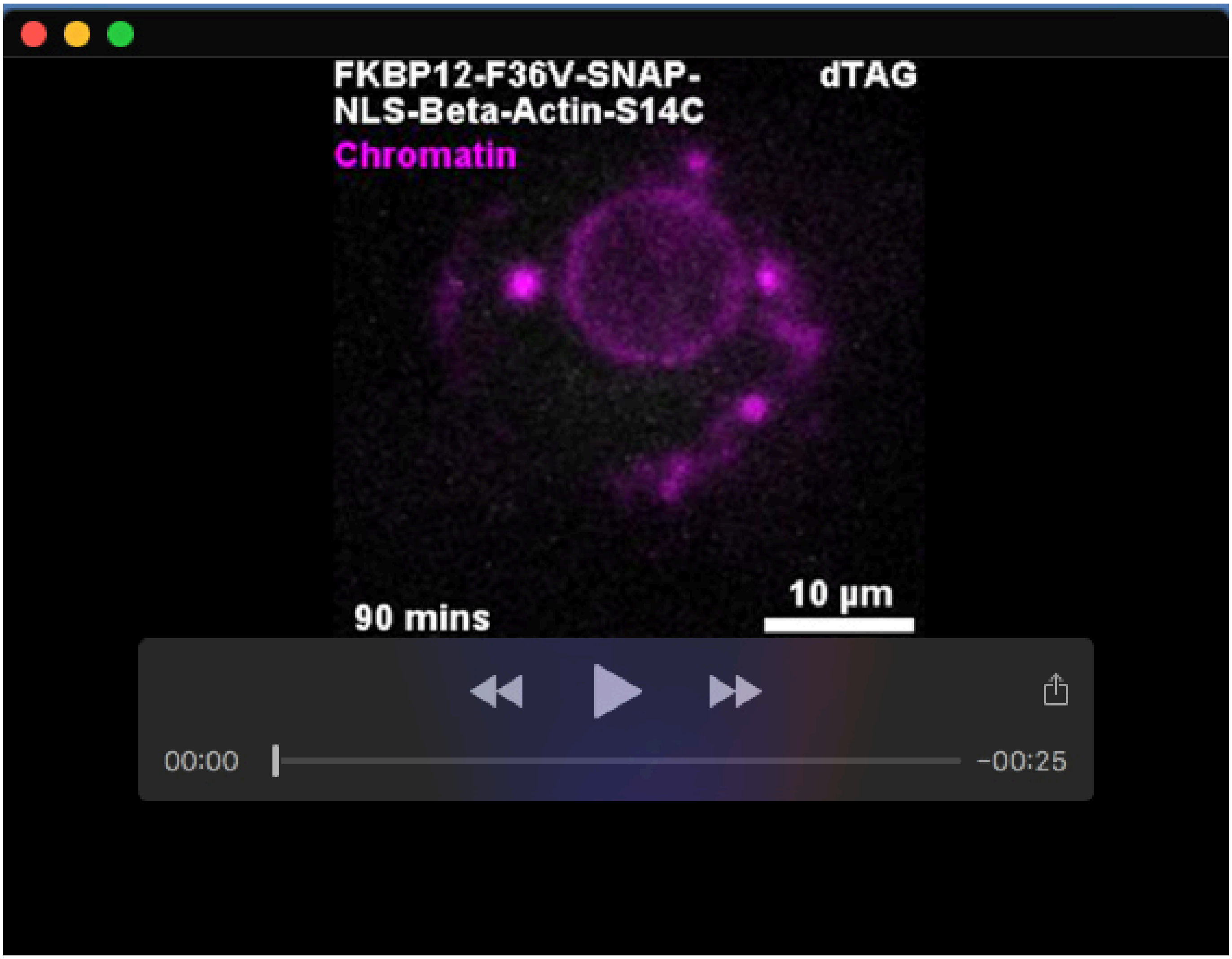

Movie 18. Time lapse movie of nuclear F-actin structures in FKBP12^{F36V}-SNAP-NLS-beta-actin-S14C expressing Dtag-13-treated mouse oocyte. Chromatin (magenta) is labelled with 5-SiR-Hoechst.

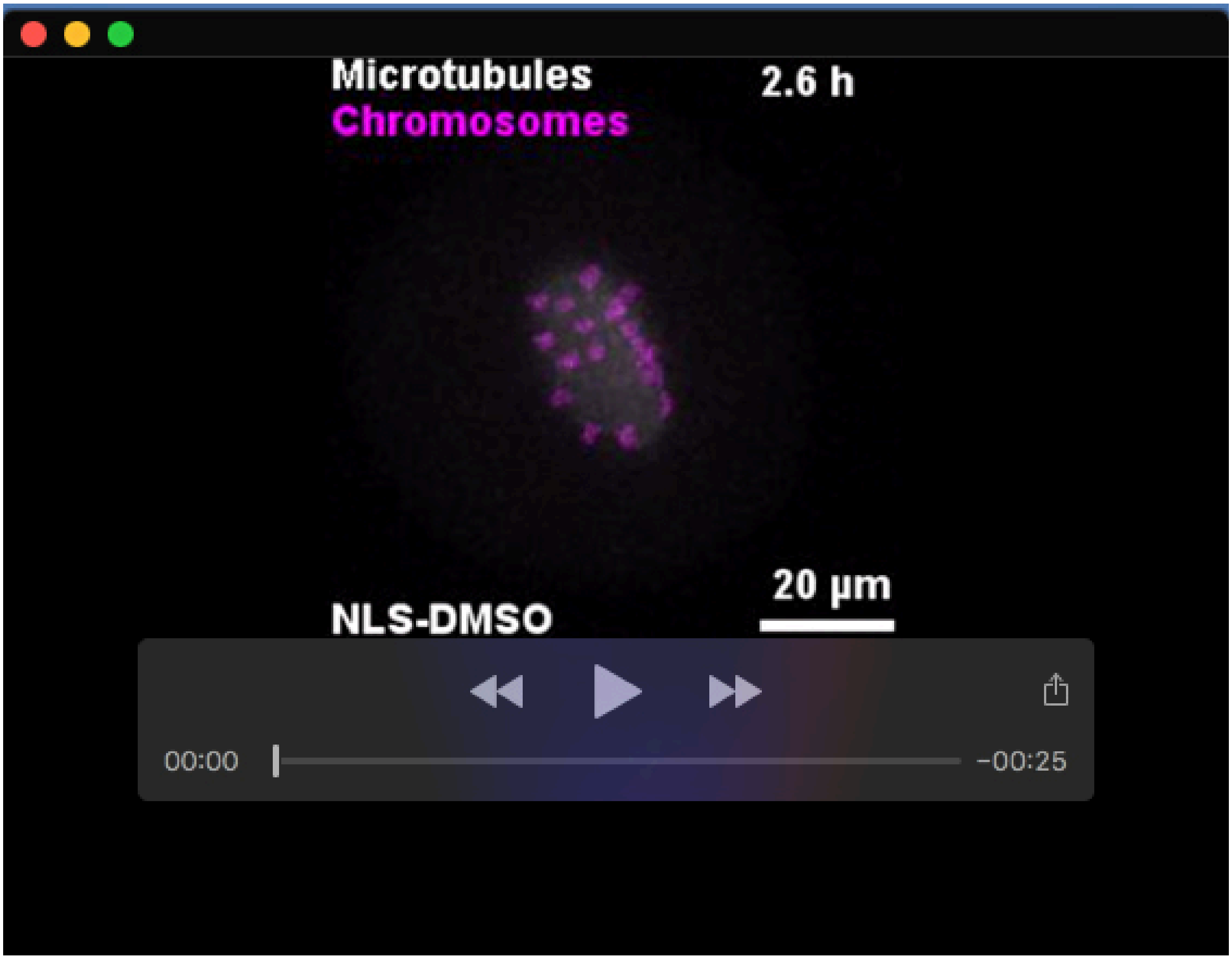

Movie 19. Time lapse movie of nuclear F-actin structures in FKBP12^{F36V}-FLAG-NLS expressing DMSO-treated mouse oocyte. Chromatin (magenta) is labelled with 5-SiR-Hoechst.

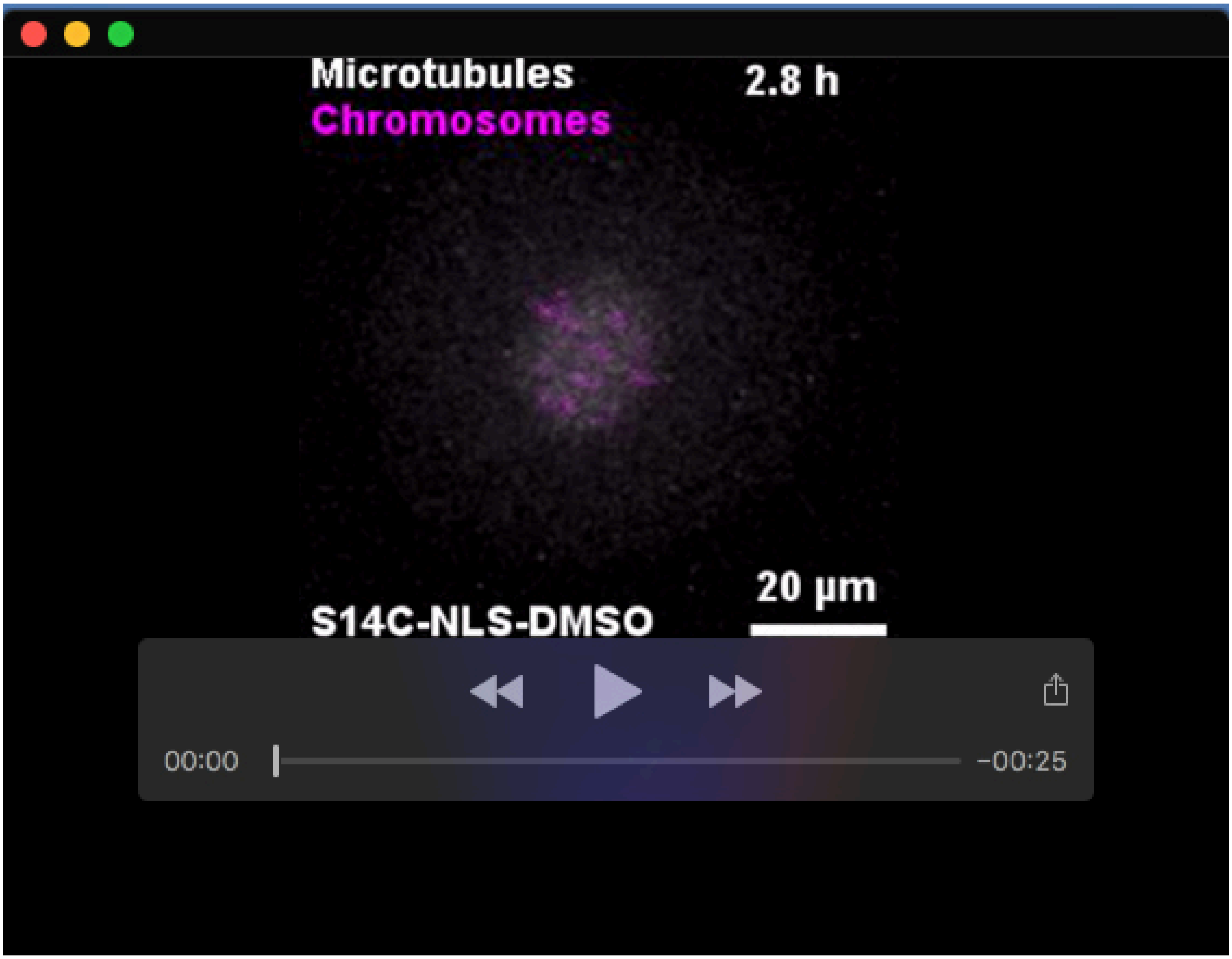

Movie 20. Time lapse movie of nuclear F-actin structures in FKBP12^{F36V}-SNAP-NLS-beta-actin-S14C expressing DMSO-treated mouse oocyte. Chromatin (magenta) is labelled with 5-SiR-Hoechst.

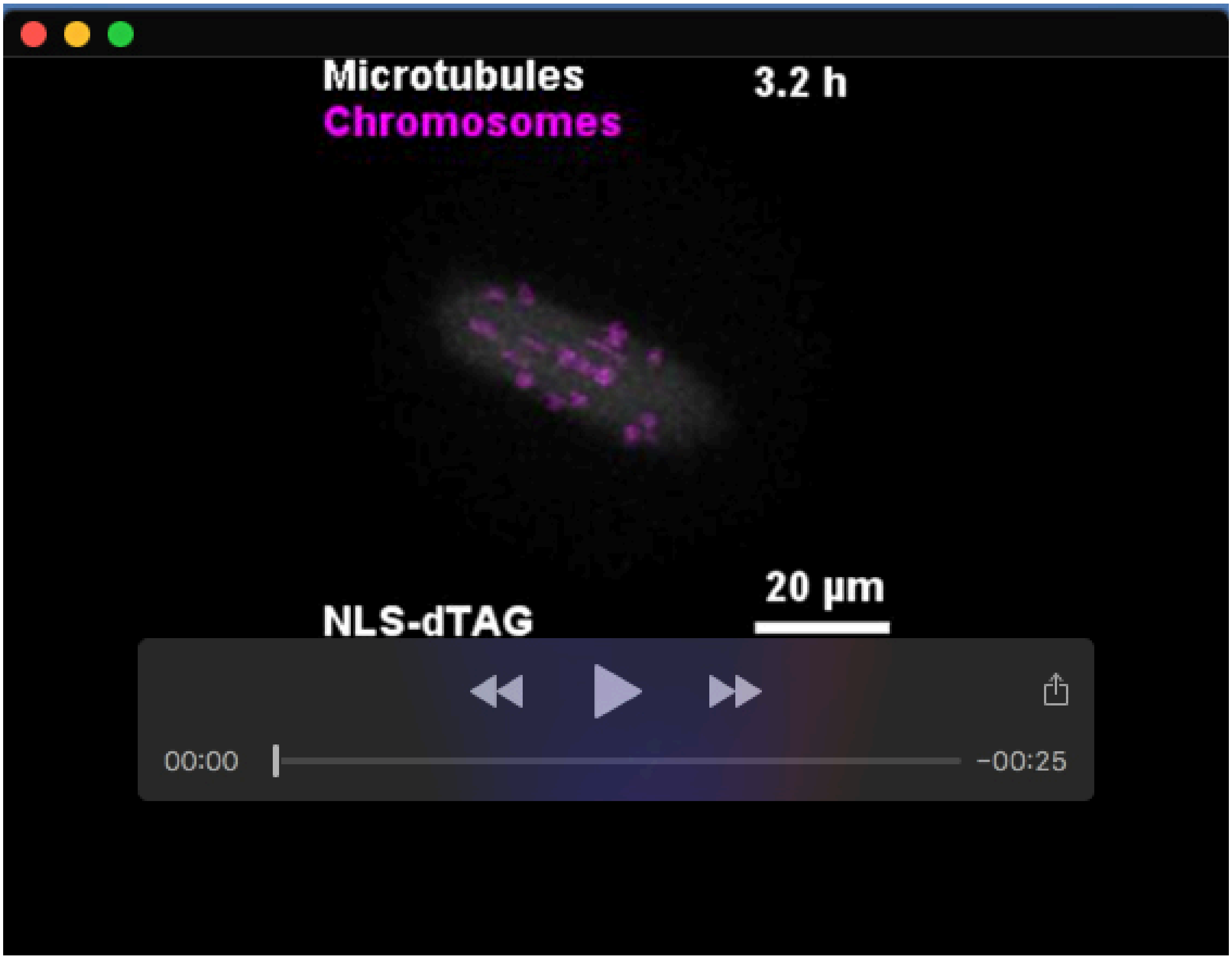

Movie 21. Time lapse movie of nuclear F-actin structures in FKBP12^{F36V}-FLAG-NLS expressing dTAG-13-treated mouse oocyte. Chromatin (magenta) is labelled with 5-SiR-Hoechst.

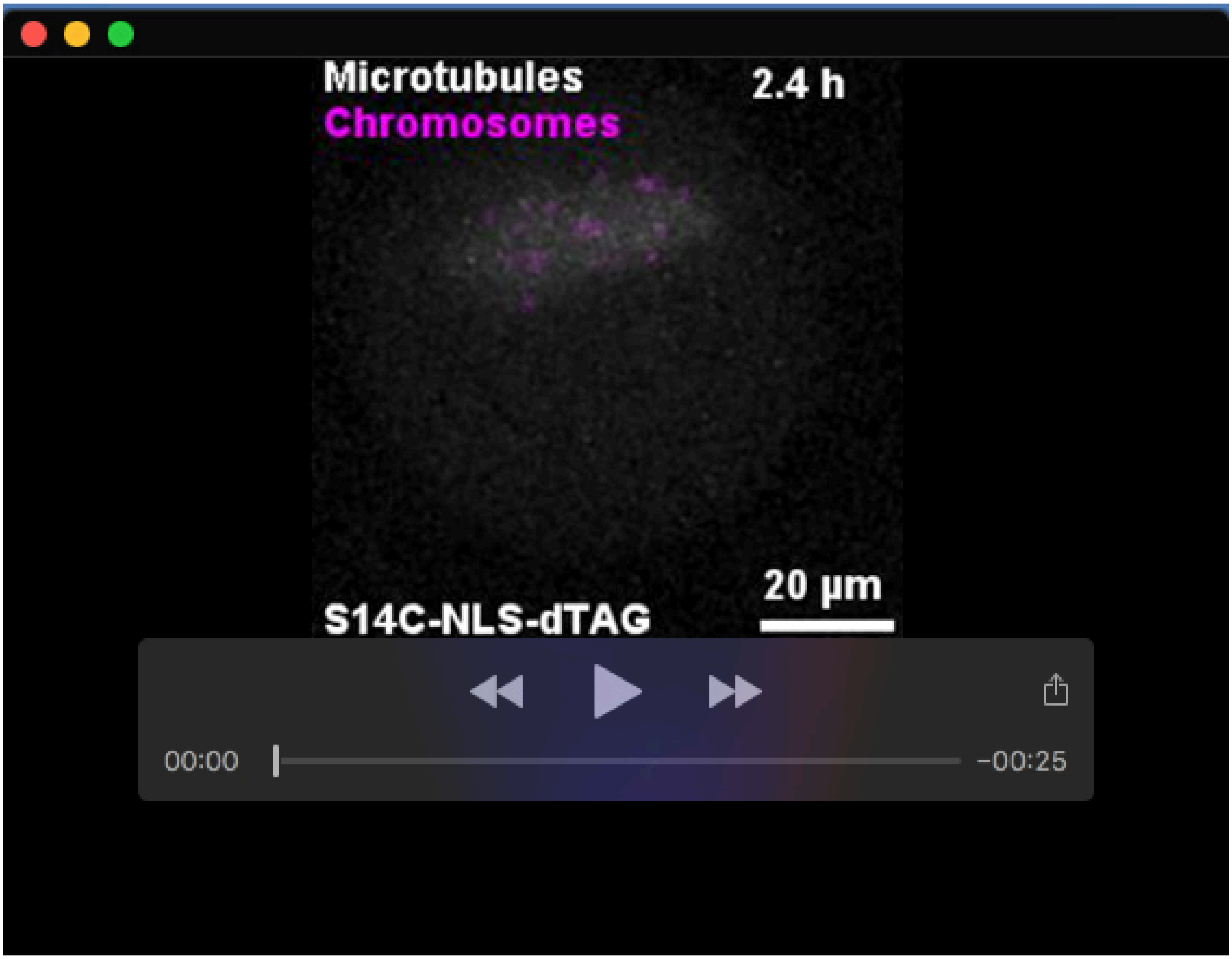

Movie 22. Time lapse movie of nuclear F-actin structures in FKBP12^{F36V}-SNAP-NLS-beta-actin-S14C expressing dTAG-13-treated mouse oocyte. Chromatin (magenta) is labelled with 5-SiR-Hoechst.
